# Prevalence, risk factors and management of pressure injuries and their implications for palliative care: A rapid overview of reviews

**DOI:** 10.1177/02692163251393817

**Published:** 2025-11-26

**Authors:** Omar Dewidar, Hind Sabri, Elizabeth Ghogomu, Shweta Jaitly, Marie-Claude Legacy, Shirley H. Bush, Jill Rice, Vivian Welch

**Affiliations:** 1 Temerty Faculty of Medicine, University of Toronto, ON, Canada; 2 Bruyère Health Research Institute, University of Ottawa, ON, Canada; 3 Department of Medicine, University of Ottawa, ON, Canada; 4 Bruyère Continuing Care, Ottawa, ON, Canada; 5 Bruyère Health Research Institute, Ottawa, ON, Canada; 6 Ottawa Hospital Research Institute, ON, Canada; 7 Department of Palliative Care, Bruyère Health, Ottawa, ON, Canada; 8 School of Epidemiology and Public Health, University of Ottawa, ON, Canada

**Keywords:** palliative care, older adults, end of life, pressure injuries, pressure ulcers, systematic review, long-term care setting

## Abstract

**Introduction:**

Evidence around pressure injuries in palliative care settings is limited and fragmented.

**Aim:**

To synthesize systematic review evidence regarding prevalence, risk factors, prevention and treatment of pressure injuries in non-acute care settings, and evaluate implications for specialist palliative care.

**Design:**

A rapid overview of reviews following the Cochrane guidance for overviews.

**Data sources:**

We searched four databases (MEDLINE, Cochrane Database of Systematic Reviews, JBI EBM Reviews, and CINAHL) from inception to October 2024. We included systematic reviews on adult patients in inpatient Palliative Care Units or settings likely involving end-of-life care.

**Results:**

Seventeen systematic reviews incorporating 149 unique studies (*n* = 5,856,293) were included. Thirteen of the reviews were high quality. Only one low-quality review specifically addressed palliative care, focusing on prevalence and risk factors. Interventions were categorized into nutritional, physical (e.g. repositioning, tilt strategies), topical/dressing-based, electrical stimulation and organizational approaches. Frequent repositioning and certain nutritional interventions may reduce incidence or surface area of pressure ulcers; however, certainty remains very low. Frequent repositioning, specific tilt strategies and specific nutritional interventions may help reduce the incidence of pressure injuries and decrease injury surface area; however certainty is very low. Other interventions have limited and inconclusive evidence and were rated low to very low certainty according to GRADE criteria due to poor trial design and population and setting variations.

**Conclusions:**

The evidence base remains weak and largely indirect, offering insufficient guidance for clinical practice in palliative care. High-quality, targeted research is needed to inform effective pressure injury prevention and management in this population.

**What is already known about the topic?**

- Evidence for pressure injury prevention and treatment is limited and of low to very low certainty due to methodological limitations in trials.

**What this paper adds**

- Despite a wide range of interventions, few studies focus specifically on patients at the end of life.
- Some repositioning techniques and nutritional strategies show potential benefit, but the supporting evidence is weak and outdated.

**Implications for practice, theory or policy**

- This review highlights the lack of high-quality, up-to-date evidence for managing pressure injury in palliative care. Addressing this gap is essential to improving end-of-life patient outcomes.

## Introduction

Pressure injuries, also known as pressure ulcers, decubitus ulcers or bedsores, are a significant health concern for patients who are immobilized or experience advanced illness.^1^ Clinical practice guidelines and expert opinions state that nearly all pressure injuries can be prevented.^2^ Once they develop, patients experience significant morbidity, pain and distress both physically and psychosocially, worsening their suffering and disrupting their comfort in their final stages of life.^3,4^

Prevalence estimates for pressure injuries range from 2.2% to 24.7%^5–7^ in hospitals, and could be even higher in long-term care^8–10^ and palliative care settings.^11–13^ Despite the widespread implementation of prevention strategies, pressure injuries remain common in non-acute care settings, where immobility, comorbidities and longer lengths of stay increase vulnerability.

Pressure injuries develop from the prolonged pressure on the skin from long periods of immobilization on a surface such as a hospital bed.^2,14^ The applied pressure impacts cellular metabolism by impeding tissue circulation, leading to tissue insufficient blood flow to the skin and subsequent ischemia.

Several populations are at increased risk of development of pressure injuries. Palliative care patients tend to be bedridden for prolonged periods of time with limited mobility, thus, are at increased risk of pressure injuries.^15,16^ The additional challenges compounded by malnutrition,^17^ and individual factors such as advanced age and multiple comorbidities^18^ increase the likelihood of injury development and presents a complex situation for managing them.

Similarly, long-term care residents often have progressive, incurable illnesses and are unable to move independently with prolonged periods of stay.^19,20^

Although several overviews summarizing evidence syntheses for specific interventions addressing pressure injuries,^21–25^ but none evaluate and compare all possible strategies for prevention and treatment or are focused on the palliative care population. Management of pressure injuries in palliative care differs from other settings.^26,27^ Management decisions must align with the goals of palliative care, focusing on pain relief while aiming to improve quality of life,^28^ rather than aggressive intervention and rehabilitation.^28,29^

Given that our scoping search identified a single review focused on palliative care settings,^11^ we expanded scope to include studies in settings where palliative care is often a consideration. Therefore, this overview evaluates the prevalence, factors associated with pressure injury development, and all possible interventions to prevent or treat pressure injuries in non-acute care settings and assess the implications for palliative care.

## Methods

This study was conducted as per the registered protocol on the PROSPERO platform (registration number: CRD42024605011). We conducted the review according to the JBI guidelines^30^ and adhered the reporting to the preferred reporting items for overviews of reviews (PRIOR)^31^ statement (Completed checklist in Appendix 1).

### Search

We electronically searched Ovid MEDLINE(R) ALL, OVID EBM Reviews (CDSR) Cochrane Database of Systematic Reviews, OVID Joanna Briggs Reviews JBI EBM Reviews and CINAHL via EBSCOhost from inception to October 2024. Searches were not limited by study design, date or language. Two seed articles^11,32^ were used to retrieve related articles in PUBMED and Scopus. Search strategies are presented in Appendix 2. An experienced librarian conducted the search using optimized term selection strategies appropriate for systematic reviews. No gray literature was searched given the rapid nature of the review.

### Eligibility

We included English language systematic reviews of adult patients (⩾18 years) admitted to inpatient Palliative Care Units (PCUs) in high-income countries (classified by the World Bank). In addition to the reviews that were clearly documented as having been conducted in palliative care settings (not limited to end of life), we also included relevant reviews whose study populations were older adults (⩾65 years of age) who were in non-acute care and likely to include individuals progressing toward end-of-life care (e.g. hospitals, nursing homes or long-term care facilities, home health agencies, hospices, community residence, home-based care).^33^ Reviews reported any of the following: the prevalence of pressure injuries, key risk and prognostic factors associated with the development and progression of pressure injuries, and the effectiveness of pharmacological and non-pharmacological interventions for the prevention and/or treatment of pressure injuries. Outcomes of interest were incidence of pressure injury development, time to complete healing, adverse events, and quality of life.

### Screening and data extraction

Two reviewers independently screened 10% of records to establish inter-rater reliability (kappa > 0.80). Disagreements were resolved by discussion. Once consistency was achieved, the remaining records were screened by a single reviewer (OD).

We developed a standardized pretested Microsoft Excel sheet to extract data, such as study year, participant characteristics, review objective, outcome measures, main findings, risk of bias and certainty in evidence assessments. Data extraction items are listed in Appendix 3. OD extracted the data and HS verified for accuracy. Disagreements were resolved by discussion.

### Review quality assessment

We used the AMSTAR 2.0 tool^34^ to assess the quality of the included reviews. The AMSTAR 2.0 tool includes 16 items addressing various aspects of the review process. These items are divided into two categories: seven critical Items that determine the robustness of the systematic review. These critical domains directly impact the credibility and reliability of the review findings. The remaining nine items are considered non-critical. They cover additional important aspects, though they are not considered essential for the overall quality assessment. Assessments were conducted by OD and HS independently. Disagreements were resolved by discussion. Based on the item responses, we assigned an overall confidence in the systematic review results as “high,” “moderate,” “low” or “critically low.”

### Overlap between reviews

We analyzed the overlap of primary studies in two or more systematic reviews to inform interpretation, and assessed the degree of overlap using the corrected covered area (CCA).^35^ The CCA is a percentage calculated by subtracting the frequency of unique studies from the frequency of all the studies (including repeated occurrences) across reviews, divided by the product of the number of reviews and total number of primary studies, subtracted by the number of unique studies. This result yields a proportion, which is converted to a percentage to indicate the degree of overlap (i.e., ⩽5%: slight overlap, 6%–10%: moderate overlap, 11%–15%: high overlap and ⩾ 15%: very high overlap). We used the GROOVE (Graphical Representation of Overlap for OVErviews) tool for this calculation and to visualize the overlap.^36^

## Results

### Study selection

**Figure 1.**
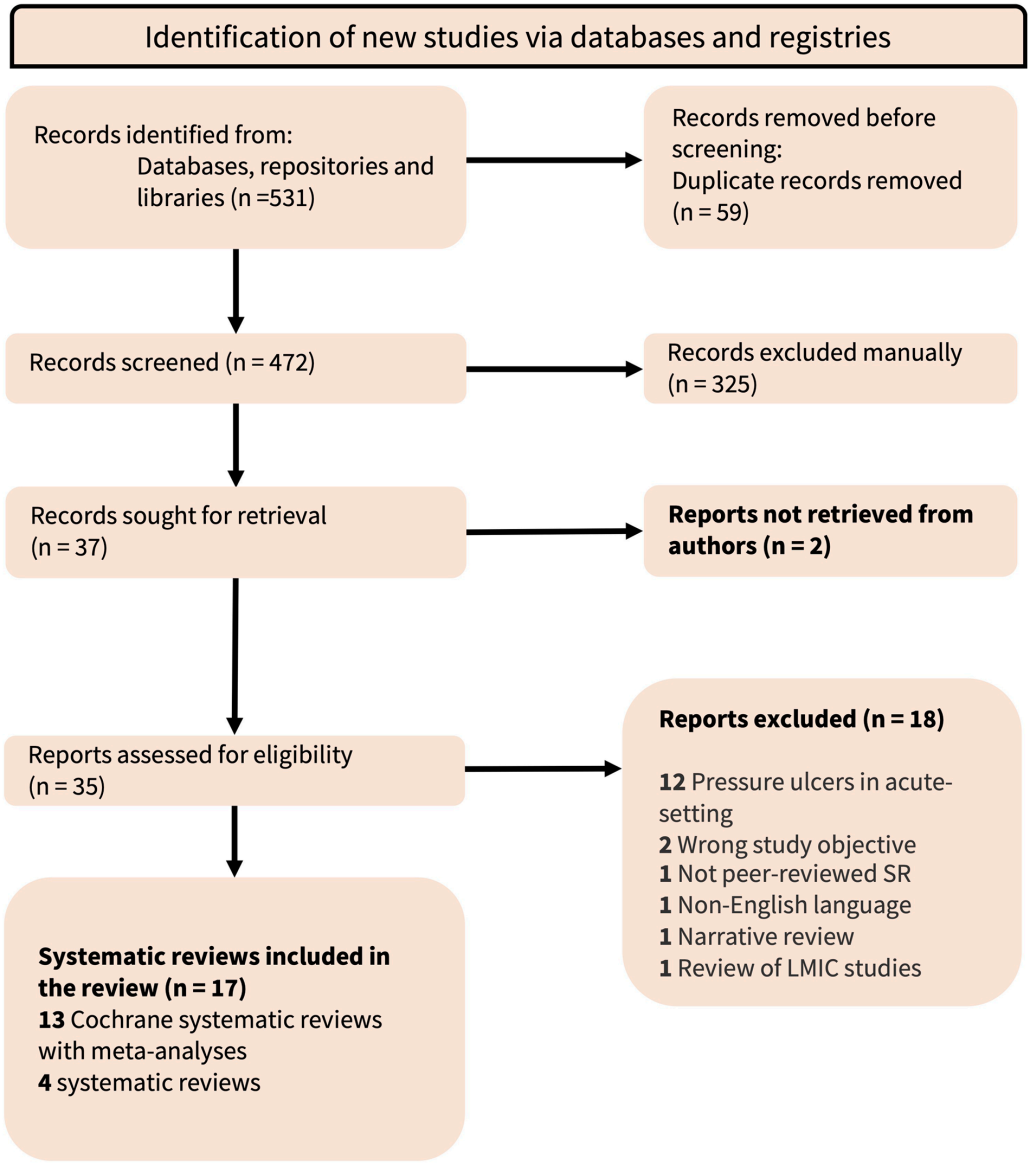
PRISMA flow chart. LMIC: low-middle-income countries.

From 531 records at title and abstract stage and 35 at full text, we included 15 systematic reviews^11,32,37–49^; 13 with meta-analyses.^32,37,39–49^ The PRISMA flow chart is displayed in Figure 1. A list of excluded reviews with reasons for exclusion is provided in Appendix 4.

### Characteristics of included systematic reviews

The included systematic reviews were published between 2009 and 2024 (Appendix 5). Eleven reviews evaluated prevention interventions^32,37–44,48,50^, five evaluated treatment^32,45–47,49^, two review assessed prevalence^11,51^ and one assessed risk factors.^11^ Only Ferris et al.^11^ focused on patients receiving palliative care and assessed risk factors and prevalence of pressure injuries. The remaining reviews focused on wider populations, so we considered only relevant primary studies. A total of 165 primary studies (149 unique studies) were included in the reviews. Thirteen reviews^32,37,39–49^ reported quality or risk of bias assessments using the Cochrane risk of bias tool^52^ and assessed certainty of evidence using GRADE.^53^ Two other reviews^50,51^ used a validated tool for assessing risk of bias in prevalence studies^54^ and JBI MASTARI criteria.^55^

### Systematic review populations

#### Participants and settings

Treatment and prevention reviews included 15,593 participants (5,840,700 participants in prevalence reviews^11,51^), with the number in each review ranging from 39^47^ to 63,907.^11^ The mean age of participants ranged from 55^39^ to 92.5^50^ years and the proportion of males ranged from 5%^49^ to 78%^32^ when reported. The most frequent setting was long-term care homes or nursing homes, followed by rehabilitation or geriatric care in various settings and hospitals. Studies were predominantly conducted in Italy, the United States, United Kingdom, Canada and Poland.

The duration of pressure injuries ranged from a mean of 4 days to over 12 months. The severity of the pressure injuries varied widely across studies, with participants most frequently having stage II pressure injuries. Anatomical location of the injuries also varied across studies with the sacrum being the most commonly affected site, followed by the coccyx, buttocks and heels.

### Quality appraisal of the included reviews

All but three of the reviews were assessed as high quality. Mäki-Turja-Rostedt et al.^50^ was rated low quality due to lack of protocol registration and not providing a list of excluded studies and justification for exclusion. Ferris et al.^11^ and Junkin and Gray^38^ were rated very low quality: primarily due to not providing a list of excluded studies and justification for exclusion, and not using a satisfactory technique for assessing the risk of bias in individual studies that were included in the respective reviews (both critical domains in AMSTAR 2.0). Details regarding the full quality appraisal assessments are in Appendix 6.

### Overlap in primary studies included in reviews

**Figure 2.**
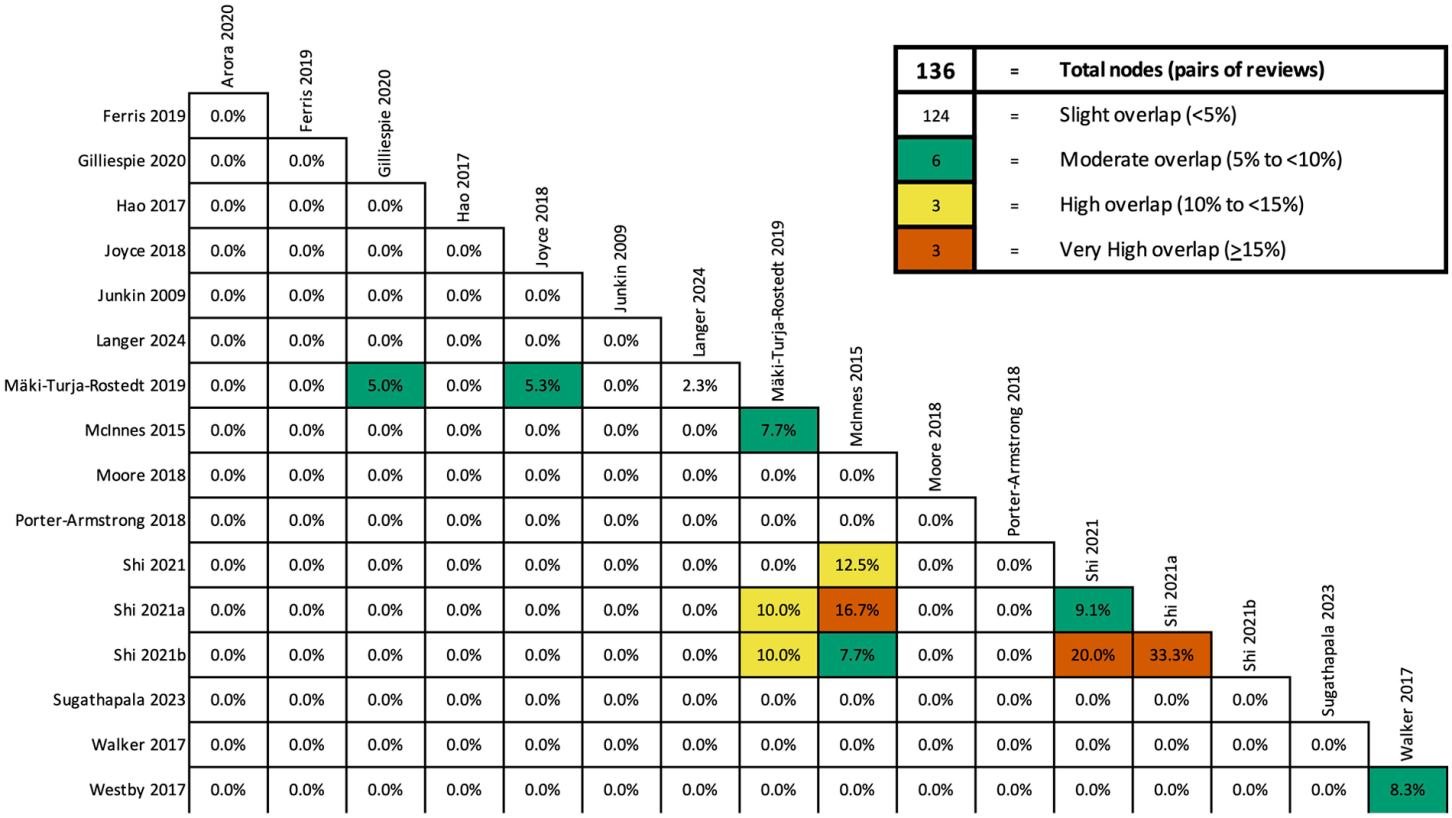
Overlap of primary studies in the review.

Sixteen primary studies were included in more than one review and the calculated CCA was 0.67%, indicating a slight overlap (Figure 2). There was overlap between McInnes et al.^39^ and the three Shi et al.^42–44^ reviews as they all evaluated the effects of support surfaces to prevent pressure injuries (CCA 7.7%–33%). There was moderate overlap between Westby et al.^45^ and Walker et al.^49^ as they both investigated the effects of different dressing agents. There was also moderate overlap between Mäki-Turja-Rostedt et al.^50^ and Langer et al.^32^ and McInnes et al.^39^ No other overlap was detected.

### Types and effects of interventions

A total of 15 reviews that assessed interventions targeting the prevention and/or treatment of pressure injuries were identified. We iteratively grouped them into the following categories: nutritional interventions, electrical stimulation, repositioning strategies, support surfaces, topical and dressing interventions and organizational strategies. The evidence on the effects of pressure injuries prevention and treatment interventions was considered indirect to populations in palliative care settings because the populations were mostly in settings other than palliative care. The details of the individual systematic review findings are in Appendices 7 and 8. We describe the results in detail in Appendix 9.

#### Prevalence and risk factors

A single review^11^ found that the prevalence of pressure injuries in palliative care population is 12.4%. Risk estimates were poorly reported, limiting our understanding of pressure injury risk. Another review^51^ of pressure injuries among older adults residing in nursing homes estimated a prevalence to be 11.6% (95% CI: 9.6%–13.7%).

#### Prevention of pressure injuries

Nutritional interventions, including protein supplementation and amino acids, showed little to no significant effect in preventing pressure injuries. The use of repositioning strategies yielded inconsistent results, with some studies suggesting a potential benefit for frequent repositioning and specific tilt angles. RCTs evaluated the effects of different support surfaces, including alternating pressure air surfaces and gel mattresses, showed varying effectiveness in reducing pressure injury incidence, but no clear improvement in time to injury development or quality of life. At the institutional level, adopting new organizational strategies that involved enhanced multidisciplinary teams and nurse training programs demonstrated uncertain effects on pressure injury prevention and healing. The overall certainty of evidence was mostly low or very low due to bias and imprecision.

#### Treatment of pressure injuries

Nutritional interventions were also evaluated for treating pressure injuries. Overall, they did not significantly improve pressure injury healing with most studies showing no meaningful effect or only a non-significant reduction in injury surface area. However, a combination arginine and micronutrients suggested a potential benefit in reducing injury size Electrical stimulation failed to result in a significant improvement in pressure injury healing or surface area reduction. Likewise, topical agents and dressings, including various creams and wound dressings, did not consistently treat pressure injuries, with some topical agents potentially increasing risk. Organizational strategies also did not significantly reduce time to complete healing and minimal quality of life benefit. The overall certainty of evidence remained low to very low due to bias and imprecision.

### Quality assessment of the primary studies

The risk of bias assessments for primary studies in each of the included reviews are detailed in Appendix 10. Thirteen of the 15 reviews assessed quality using the Cochrane Risk of Bias Tool. Sugathapala et al.^51^ used a validated tool for assessing risk of bias in prevalence studies.^54^ Mäki-Turja-Rostedt et al.^50^ assessed the quality of the studies using JBI MASTARI criteria.^55^ Overall, the majority of the primary studies were at high risk of bias due to limitations with blinding participants and personnel, sampling methodology and outcome assessment. Ferris et al.^11^ did not use a validated tool. They noted that all 12 included studies were retrospective observational studies, had unclear or missing follow-up periods and unclear reporting of statistical analyses. None of the analyses were adjusted for baseline differences. Junkin and Gray^38^ did not conduct a quality assessment of primary studies.

## Discussion

Overall, there was no convincing evidence supporting any specific intervention for the prevention or treatment of pressure injuries in older adults in non-acute settings. We found that the evidence was methodologically weak, based on a small number of participants, and often indirect to palliative care settings. Given that the evidence on effectiveness of prevention and treatment interventions was mainly indirect, we describe in Box 1 the implications of the review findings on patients in palliative care settings.

**Box 1.** Practical recommendations for clinicians working in palliative care.

• **Nutritional interventions:** Evidence suggests protein supplementation to be ineffective for prevention but is inconclusive for treatment. In palliative care, practicality is by swallowing difficulties, pill burden, and common symptoms such as poor appetite and nausea. Implementation should be individualized, taking into account patient tolerance, comfort and feasibility in the clinical context. • **Electrical stimulation:** Evidence does not support its use, and practical barriers such as complexity and contraindications make it unsuitable for palliative patients. Clinicians should avoid electrical stimulation unless strong future evidence emerges. • **Repositioning strategies:** Repositioning every 3 h may reduce incidence more effectively than every 4 h while more frequent repositioning results may vary. In palliative care, repositioning plans should be Individualize, aiming to prevent skin breakdown while minimizing pain, breathlessness and hemodynamic compromise. Most importantly, repositioning practices should respect patient preferences. • **Advanced support surfaces:** These may be effective for delaying injury development but do not replace repositioning. Clinicians should select surfaces that provide adequate pressure redistribution, shear reduction and optimize microclimate control, while ensuring patient comfort. • **Topical Agents and dressings:** Avoid DSMO (dimethyl sulfoxide) creams due to increased risks. Some foam dressings may reduce risk of pressure injury development, but implementation should be tailored to patient comfort, wound-related symptoms and goals of care. • **Organizational strategies:** Interventions at the organization level have shown neutral or adverse effects suggesting caution in implementation. Training should prioritize improving clinicians’ practical skills and reinforce fundamental bedside care, rather than relying on top-down policy changes.

Nutritional interventions such as arginine and micronutrient diet may reduce pressure injury surface area, but evidence remains inconclusive. In palliative care, adherence to these interventions may be challenging^56^ given common issues such as swallowing difficulties and pill burden of the supplements, and poor appetite.^57^ Similarly, electrical stimulation lacks evidence for treatment and its complexity reduces its practicality in this context.^58^ In contrast, advanced support surfaces, such as alternating pressure air mattresses, appear to delay injury development but lack robust data on healing outcomes and quality of life. Repositioning is still needed and support surfaces should be selected based on a patient’s need for microclimate control, shear reduction, pressure redistribution, turn and assist.^59^

The data on topical agents and dressings is mixed with several studies showing non-significant findings with low certainty of evidence. Notably, DSMO creams have been associated with a significantly increased risk of pressure injury development and should therefore be avoided. In current clinical practice guidelines, the use of foam dressings over bony prominences is recommended for prevention of pressure injuries in the context of palliative care.^59^ Patient’s comfort and needs should be prioritized prior to their implementation. Interventions involving multidisciplinary teams show neutral to potentially adverse effects, raising concerns about the dilution of bedside care responsibilities. However, training programs for bedside caregivers show promise in improving outcomes. The latest clinical practice guidelines recommend organizations equip healthcare workers in the patient’s circle of care with the appropriate skills and knowledge of pressure injuries to provide the best care possible.^58,59^

## Gaps in the evidence

We identified only one low quality systematic review focused on patients receiving palliative care that assessed risk factors and the prevalence of pressure injuries. No reviews for prevention or treatment were focused on palliative care patients. The majority of the evidence in the reviews, even within the review focused on palliative care, was indirectly applicable to palliative care settings. The values and priorities of care may overlap but tend to be distinct from those of long-term care homes and hospital care. Furthermore, important outcomes such as quality of life and adverse events were rarely assessed in trials, leaving a critical gap in understanding the impact of the interventions on patients’ well-being and how they can support easing their pain and distress at end of life.

The majority of the evidence was from small studies with serious limitations in study design conduct and delivery which significantly impacted the certainty in the evidence. Furthermore, the majority of the randomized controlled trials (RCTs) on this topic are more than 20 years old. This has significant implications on the applicability of the results given the changes over the years including increased focus on providing palliative care to patients with non-cancer conditions,^60,61^ a growing aging population^62^ and clinical practice guidelines recommending early palliative care with active treatment.^63^ In addition, a number of the dressings and topical agents used in the RCTs may no longer be available or accepted as part of current nursing practice and local clinical guidelines. Clinical practice for the care of pressure injuries continues to evolve with the availability of more advanced dressings and newer topical agents. Thus, there is an urgent need for up-to-date reviews and additionally well-designed RCTs.

## Strengths and limitations

To our knowledge, this is the first overview to identify and synthesize the existing systematic review evidence on prevalence, risk factors and interventions to prevent and treat pressure injuries in palliative care settings. We conducted this overview using rigorous and transparent methods according to the JBI guidelines and the reporting of this overview adheres to the PRIOR guidelines. Almost all the evidence was assessed for risk of bias and are accompanied by GRADE assessments. There was minimal overlap between the reviews, accurately reflecting the effects of the interventions.

The inherent nature of this rapid overview is that it focused only on published systematic reviews, so primary studies published after the latest review search dates would have not been included and may be informative. We did not include gray literature searches which may have limited the comprehensiveness of our findings. We also restricted inclusion to systematic reviews in English language and conducted in high-income countries. Thus, the applicability of the findings to settings in lower- or middle-income countries remains unclear.

Our conclusions regarding the effectiveness of interventions to treat and prevent pressure injuries were extrapolated from indirect evidence as individuals receiving palliative care were either a minority within the study populations (e.g. most rehabilitation unit patients do not die) or had limited exposure time (e.g. most time spent in long-term care, nursing homes, or home care is spent living rather than dying). However, given that these trials included individuals with significant illness, they were more likely to require a palliative care approach compared to patients in acute or intensive care settings, which we excluded.

## Future research

Given the broad types of interventions to address our research question, there is a need to update almost all the Cochrane systematic reviews, as many are outdated by at least 3 years. This is particularly important for interventions such as repositioning strategies and advanced pressure support surfaces, which have shown promising effects despite the low certainty of evidence. Future research on pressure injuries should consider distinguishing between different settings more explicitly, as the appropriateness of a specific intervention over another may vary depending on the context.

Our overview suggests there is potential for advanced support surfaces, such as alternating pressure air mattresses in reducing pressure injuries. In practice, it would be ideal to use the latest evidence-based proven technology, but availability and costs may limit its use. The focus of future research should extend beyond the choice of mattress to include the materials and practices employed on these surfaces. For example, the continued use of multiple layers of linen and incontinence pads, directly beneath patients remains common in palliative care settings.^59,64^ Lastly, healthcare provider training interventions should be refined to ensure foundational care remains prompt and focuses on improving quality of life and comfort for palliative care patients.

Additionally, future trials should prioritize evaluating high-energy and high-protein diets in palliative care populations as adequate protein consumption has been linked to lower rates of malnutrition, improved treatment outcomes, and longer survival.^65^

To address the current risk of bias limitations in the evidence-base, well-designed RCTs are needed. These trials should prioritize assessing feasibility and implementation outcomes, ensuring that interventions are adhered to and are scalable in real-world settings. Additionally, all the reviews focused on single-component interventions which highlights a gap in exploring the effectiveness of multi-component interventions. Several review authors noted that quality of life was not assessed in the trials which is an important outcome for palliative care.

Finally, a major gap in the current literature is the lack of quality-of-life outcomes. Future primary studies should prioritize these outcomes as they are central to palliative care. There is also a need to develop a core outcome set for future intervention studies for pressure injuries in palliative care populations.^66^

## Conclusion

Our overview highlights that evidence for prevalence, risk factors and interventions to prevent and treat pressure injuries is limited in both quality and quantity, primarily due to poor trial design and implementation. Almost all the evidence was rated low or very low certainty. A small subset of the evidence directly applies to the palliative care population. Healthcare providers and their managers should consider the benefits of each intervention for palliative care patients, ensuring that interventions align with the person’s goals of care and values.

## Supplemental Material

sj-docx-1-pmj-10.1177_02692163251393817 – Supplemental material for Prevalence, risk factors and management of pressure injuries and their implications for palliative care: A rapid overview of reviews Supplemental material, sj-docx-1-pmj-10.1177_02692163251393817 for Prevalence, risk factors and management of pressure injuries and their implications for palliative care: A rapid overview of reviews by Omar Dewidar, Hind Sabri, Elizabeth Ghogomu, Shweta Jaitly, Marie-Claude Legacy, Shirley H. Bush, Jill Rice and Vivian Welch in Palliative Medicine
