# Supplementary material for: Prevalence, risk factors and management of pressure injuries and their implications for palliative care: A rapid overview of reviews
Source: Palliat Med. 2025 Nov 26;40(1):21–30. doi: 10.1177/02692163251393817 (PMC12779764; doi:10.1177/02692163251393817)
Supplement: sj-docx-1-pmj-10.1177_02692163251393817 – Supplemental material for Prevalence, risk factors and management of pressure injuries and their implications for palliative care: A rapid overview of reviews [file sj-docx-1-pmj-10.1177_02692163251393817.docx]

**Title:** Prevalence, risk factors and management of pressure injuries and their implications for palliative care: A rapid overview of reviews

Table of Contents

[Appendix 1. PRIOR statement 2](#_Toc208826296)

[Appendix 2 – Electronic search strategies 5](#_Toc208826297)

[Appendix 3. Data extraction items. 8](#_Toc208826298)

[Appendix 4. List of excluded studies. 9](#_Toc208826299)

[Appendix 5. Characteristics of the included systematic reviews. 11](#_Toc208826300)

[Appendix 6. Methodological quality of the reviews assessed by AMSTAR 2.0 tool. 17](#_Toc208826301)

[Appendix 7. Treatment and prevention of pressure injuries organized by outcome. 19](#_Toc208826302)

[Appendix 8. Categorization of treatment and prevention of pressure injuries by types of interventions. 37](#_Toc208826303)

[Appendix 9. Detailed description of the results including the most effective interventions. 52](#_Toc208826304)

[Risk factors for pressure injuries 52](#_Toc208826305)

[Prevalence of pressure injuries 52](#_Toc208826306)

[Prevention of pressure injuries 52](#_Toc208826307)

[Treatment of pressure injuries 53](#_Toc208826308)

[Appendix 10. Risk of bias of primary studies in the included systematic reviews. 56](#_Toc208826309)

## Appendix 1. PRIOR statement

| **Section**  Topic | | **#** | | **Item** | **Location reported** |
| --- | --- | --- | --- | --- | --- |
| **TITLE** | | | | |  |
| Title | | 1 | | Identify the report as an overview of reviews. | 1 |
| **ABSTRACT** | | | | |  |
| Abstract | | 2 | | Provide a comprehensive and accurate summary of the purpose, methods, and results of the overview of reviews. | 3 |
| **INTRODUCTION** | | | | |  |
| Rationale | | 3 | | Describe the rationale for conducting the overview of reviews in the context of existing knowledge. | 4 |
| Objectives | | 4 | | Provide an explicit statement of the objective(s) or question(s) addressed by the overview of reviews. | 4 |
| **METHODS** | | | | |  |
| Eligibility criteria | | 5a | | Specify the inclusion and exclusion criteria for the overview of reviews. If supplemental primary studies were included, this should be stated, with a rationale. | 5 |
|  |  | 5b | | Specify the definition of ‘systematic review’ as used in the inclusion criteria for the overview of reviews. | PROSPERO Registration |
| Information sources | | 6 | | Specify all databases, registers, websites, organizations, reference lists, and other sources searched or consulted to identify systematic reviews and supplemental primary studies (if included).  Specify the date when each source was last searched or consulted. | 5 |
| Search strategy | | 7 | | Present the full search strategies for all databases, registers and websites, such that they could be reproduced. Describe any search filters and limits applied. | 5 |
| Selection process | | 8a | | Describe the methods used to decide whether a systematic review or supplemental primary study (if included) met the inclusion criteria of the overview of reviews. | 5 |
|  |  | 8b | | Describe how overlap in the populations, interventions, comparators, and/or outcomes of systematic reviews was identified and managed during study selection. | 5 |
| Data collection process | | 9a | | Describe the methods used to collect data from reports. | 5 |
|  |  | 9b | | If applicable, describe the methods used to identify and manage primary study overlap at the level  of the comparison and outcome during data collection. For each outcome, specify the method used to illustrate and/or quantify the degree of primary study overlap across systematic reviews. | 6 |
|  |  | 9c | | If applicable, specify the methods used to manage discrepant data across systematic reviews during data collection. | NA |
| Data items | | 10 | | List and define all variables and outcomes for which data were sought. Describe any assumptions made and/or measures taken to identify and clarify missing or unclear information. | 5, supplementary material S3 |
| Risk of bias assessment | | 11a | | Describe the methods used to *assess* risk of bias or methodological quality of the included systematic reviews. | 5 |
|  |  | 11b | | Describe the methods used to *collect* data on (from the systematic reviews) and/or *assess* the risk of bias of the primary studies included in the systematic reviews. Provide a justification for instances where flawed, incomplete, or missing assessments are identified but not re-assessed. | 5 |
|  |  | 11c | | Describe the methods used to *assess* the risk of bias of supplemental primary studies (if included). | 5 |
| Synthesis methods | | 12a | | Describe the methods used to summarize or synthesize results and provide a rationale for the choice(s). | PROSPERO Registration |
|  |  | 12b | | Describe any methods used to explore possible causes of heterogeneity among results. | NA |
|  |  | 12c | | Describe any sensitivity analyses conducted to assess the robustness of the synthesized results. | NA |
| Reporting bias assessment | | 13 | | Describe the methods used to *collect* data on (from the systematic reviews) and/or *assess* the risk of bias due to missing results in a summary or synthesis (arising from reporting biases at the levels of the systematic reviews, primary studies, and supplemental primary studies, if included). | 5 |
| Certainty assessment | | 14 | | Describe the methods used to *collect* data on (from the systematic reviews) and/or *assess* certainty (or confidence) in the body of evidence for an outcome. | 5 |
| **RESULTS** | | | | |  |
| Systematic review and supplemental primary study selection | | 15a | | Describe the results of the search and selection process, including the number of records screened, assessed for eligibility, and included in the overview of reviews, ideally with a flow diagram. | 6 |
|  |  | 15b | | Provide a list of studies that might appear to meet the inclusion criteria, but were excluded, with the main reason for exclusion. | 6 |
| **Section**  Topic | | # | | Item | Location reported |
| Characteristics of systematic reviews and supplemental primary studies | | 16 | | Cite each included systematic review and supplemental primary study (if included) and present its characteristics. | 6 |
| Primary study overlap | | 17 | | Describe the extent of primary study overlap across the included systematic reviews. | 7 |
| Risk of bias in systematic reviews, primary studies, and  supplemental primary studies | | 18a | | Present assessments of risk of bias or methodological quality for each included systematic review. | 11; Supplementary material S5 |
|  |  | 18b | | Present assessments (collected from systematic reviews or assessed anew) of the risk of bias of the primary studies included in the systematic reviews. | Supplementary material S9 |
|  |  | 18c | | Present assessments of the risk of bias of supplemental primary studies (if included). | Supplementary material S9 |
| Summary or synthesis of results | | 19a | | For all outcomes, summarize the evidence from the systematic reviews and supplemental primary studies (if included). If meta-analyses were done, present for each the summary estimate and its precision and measures of statistical heterogeneity. If comparing groups, describe the direction of the effect. | Supplementary material S8 |
|  |  | 19b | | If meta-analyses were done, present results of all investigations of possible causes of heterogeneity. | NA |
|  |  | 19c | | If meta-analyses were done, present results of all sensitivity analyses conducted to assess the robustness of synthesized results. | NA |
| Reporting biases | | 20 | | Present assessments (collected from systematic reviews and/or assessed anew) of the risk of bias due to missing primary studies, analyses, or results in a summary or synthesis (arising from reporting biases at the levels of the systematic reviews, primary studies, and supplemental primary  studies, if included) for each summary or synthesis assessed. | NA |
| Certainty of evidence | | 21 | | Present assessments (collected or assessed anew) of certainty (or confidence) in the body of evidence for each outcome. | 8-11, Supplementary material S6 and S7 |
| **DISCUSSION** | | | |  |  |
| Discussion | | 22a | Summarize the main findings, including any discrepancies in findings across the included systematic reviews and supplemental primary studies (if included). | 12 |  |
|  |  | 22b | Provide a general interpretation of the results in the context of other evidence. | 12 |  |
|  |  | 22c | Discuss any limitations of the evidence from systematic reviews, their primary studies, and supplemental primary studies (if included) included in the overview of reviews. Discuss any limitations of the overview of reviews methods used. | 13 |  |
|  |  | 22d | Discuss implications for practice, policy, and future research (both systematic reviews and primary research). Consider the relevance of the findings to the end users of the overview of reviews, e.g., healthcare providers, policymakers, patients, among others. | 12-13 |  |
| **OTHER INFORMATION** | | | |  |  |
| Registration and protocol | | 23a | Provide registration information for the overview of reviews, including register name and registration number, or state that the overview of reviews was not registered. | 6 |  |
|  |  | 23b | Indicate where the overview of reviews protocol can be accessed, or state that a protocol was not prepared. | 6 |  |
|  |  | 23c | Describe and explain any amendments to information provided at registration or in the protocol. Indicate the stage of the overview of reviews at which amendments were made. | NA |  |
| Support | | 24 | Describe sources of financial or non-financial support for the overview of reviews, and the role of the funders or sponsors in the overview of reviews. | 14 |  |
| Competing interests | | 25 | Declare any competing interests of the overview of reviews' authors. | 14 |  |
| Author information | | 26a | Provide contact information for the corresponding author. | 1 |  |
|  |  | 26b | Describe the contributions of individual authors and identify the guarantor of the overview of reviews. | 14 |  |
| Availability of data and other materials | | 27 | Report which of the following are available, where they can be found, and under which conditions they may be accessed: template data collection forms; data collected from included systematic reviews and supplemental primary studies; analytic code; any other materials used in the overview of reviews. | NA |  |

## Appendix 2 – Electronic search strategies

Note: Note: Searches were conducted using an Ovid multi-database.

**Database: Ovid MEDLINE(R) ALL <1946 to October 17, 2024>, Cochrane Database of Systematic Reviews, JBI EBM REviews,**

Filename Search Strategy:

Database: Ovid MEDLINE(R) ALL <1946 to October 17, 2024>

Search Strategy:

1 exp Pressure Ulcer/ (14538)

2 (pressure adj (ulcer* or sore* or injury)).mp. (19366)

3 (decubitus adj (ulcer* or sore* or injury)).mp. (2129)

4 (bed adj (ulcer* or sore* or injury)).mp. (325)

5 or/1-4 (20158)

6 exp Palliative Care/ (65901)

7 exp Terminal Care/ (58822)

8 exp Terminally Ill/ (6873)

9 palliat*.mp. (123284)

10 (terminal* adj5 (care or caring)).mp. (35881)

11 ((advanced or terminal) adj5 (ill* or disease*)).mp. (73066)

12 (end stage or end of life or last year of life or LYOL or life's end).mp. (127366)

13 or/6-12 (332158)

14 exp Inpatients/ (31689)

15 Hospices/ (5992)

16 Hospitals/ (105961)

17 ((hospital* or inpatient*) adj2 (base* or care or center* or centre* or interven* or management or model* or nurs* or program* or service* or team* or therap* or treat*)).mp. (403476)

18 hospice*.mp. (22840)

19 or/14-18 (530892)

20 5 and 13 and 19 (111)

JBI (26)

CDSR (22)

**CINAHL – EbscoHOST 1980-Oct 17, 2024**

| **#** | **Query** | **Results** |  |
| --- | --- | --- | --- |
| S20 | S5 AND S12 AND S19 | 113 |  |
|  |  |  |  |
|  |  |  |  |
| S19 | S13 OR S14 OR S15 OR S16 OR S17 OR S18 | 303,132 |  |
|  |  |  |  |
|  |  |  |  |
| S18 | (MH "Hospices") | 3,385 |  |
|  |  |  |  |
|  |  |  |  |
| S17 | (MH "Hospice Nurses") OR (MH "Hospice Patients") OR (MH "Hospice Nursing") OR (MH "Hospice Care") | 15,588 |  |
|  |  |  |  |
|  |  |  |  |
| S16 | ((hospital* or inpatient*) N2 (base* or care or center* or centre* or interven* or management or model* or nurs* or program* or service* or team* or therap* or treat*)) | 159,265 |  |
|  |  |  |  |
|  |  |  |  |
| S15 | (MH "Inpatients") | 88,904 |  |
|  |  |  |  |
|  |  |  |  |
| S14 | (MH "Hospitals") | 68,155 |  |
|  |  |  |  |
|  |  |  |  |
| S13 | (MH "Hospital Units") OR (MH "Hospital Programs") | 15,817 |  |
|  |  |  |  |
|  |  |  |  |
| S12 | S6 OR S7 OR S8 OR S9 OR S10 OR S11 | 100,474 |  |
|  |  |  |  |
|  |  |  |  |
| S11 | TI ( (end stage or end of life or last year of life or LYOL or life's end) ) OR AB ( (end stage or end of life or last year of life or LYOL or life's end) ) | 46,737 |  |
|  |  |  |  |
|  |  |  |  |
| S10 | TI ( ((advanced or terminal) N2 (ill* or disease*)) ) OR AB ( ((advanced or terminal) N2 (ill* or disease*)) ) | 14,013 |  |
|  |  |  |  |
|  |  |  |  |
| S9 | TI ( (terminal* N2 (care or caring)) ) OR AB ( (terminal* N2 (care or caring)) ) | 1,706 |  |
|  |  |  |  |
|  |  |  |  |
| S8 | TI palliat* AND AB palliat* | 14,558 |  |
|  |  |  |  |
|  |  |  |  |
| S7 | (MH "Terminally Ill Patients") | 12,628 |  |
|  |  |  |  |
|  |  |  |  |
| S6 | (MH "Palliative Care") OR (MH "Palliative Care Nursing") | 44,288 |  |
|  |  |  |  |
|  |  |  |  |
| S5 | S1 OR S2 OR S3 OR S4 | 24,248 |  |
|  |  |  |  |
|  |  |  |  |
| S4 | (decubitus N2 (ulcer* or sore* or injury)) | 488 |  |
|  |  |  |  |
|  |  |  |  |
| S3 | (bed N2 (ulcer* or sore* or injury)) | 1,672 |  |
|  |  |  |  |
|  |  |  |  |
| S2 | (pressure N2 (ulcer* or sore* or injury)) | 22,788 |  |
|  |  |  |  |
|  |  |  |  |
| S1 | (MH "Pressure Ulcer+") | 16,240  Bottom of Form |  |
|  |  |  |  |

## Appendix 3. Data extraction items.

- Name and year of study (ID)
- Type of review (systematic, scoping)
- For each review, total number of studies
- prevalence outcome reported
- Risk/prognostic factors (as appropriate)
- aim of review
- date of search
- inclusion/exclusion criteria
- number of participants
- countries
- risk/prognostic factor details
- relevant outcomes assessed and analysis details
- narrative summary findings or summary statistics
- risk of bias and certainty of evidence assessment methods conducted by authors
- author-reported limitations

## Appendix 4. List of excluded studies.

| Title | Exclusion reason |
| --- | --- |
| Management of chronic pressure ulcers: an evidence-based analysis | Not peer reviewed SR |
| The Incidence of Pressure Ulcers and its Associations in Different Wards of the Hospital: A Systematic Review and Meta-Analysis | Pressure ulcers in hospital wards |
| Risk factors for pressure injuries among critical care patients: A systematic review | Pressure ulcers in acute setting |
| Prevalence of pressure ulcers in long-term care: a global review | Could not retrieve; requested paper on researchgate; |
| Systematic review: Incidence and prevalence of mucous membrane pressure injury in adults admitted to acute hospital settings | Pressure ulcers in acute setting |
| Efficacy of Recombinant Human Epidermal Growth Factor in Pressure Injury Healing: Evidence from Chinese Randomized Controlled Trials | LMIC |
| Comprehensive Management of Pressure Injury: A Review | Wrong study design |
| Pressure ulcers in intensive care patients: a review of risks and prevention | Pressure ulcers in acute setting |
| Global prevalence and incidence of pressure injuries in hospitalised adult patients: A systematic review and meta-analysis | Pressure ulcers in acute setting |
| Risk factors for pressure ulcer development in Intensive Care Units: A systematic review | Pressure ulcers in acute setting |
| The prevalence of pressure ulcers in Europe, what does the European data tell us: a systematic review | Could not retrieve; requested full text from researchgate |
| Risk assessment tools for the prevention of pressure ulcers | Pressure ulcers in acute setting |
| Anabolic steroids for treating pressure ulcers | Pressure ulcers in acute setting |
| Nurses' pressure ulcer related judgements and decisions in clinical practice: a systematic review | Wrong study objective |
| A systematic review of prevalence and incidence of pressure ulcers/injuries in hospital emergency services | Pressure ulcers in acute setting |
| Pressure ulcer prevalence and incidence in intensive care patients: a literature review | Pressure ulcers in acute setting |
| Skin status for predicting pressure ulcer development: A systematic review and meta-analyses | Prognosis review |
| Negative pressure wound therapy for treating pressure ulcers | Pressure ulcers in acute setting |
| [The Epidemiology of Pressure Ulcer in Germany: Systematic Review] | Not in English |
| Pressure Ulcers Prevalence in the Acute Care Setting: A Systematic Review, 2000-2015 | Pressure ulcers in acute setting |

## Appendix 5. Characteristics of the included systematic reviews.

| Review | Objective | Number of eligible studies | Country(ies) | Databases searched | Search years | Interventions | Sample characteristics | Outcomes of interest | Funding |
| --- | --- | --- | --- | --- | --- | --- | --- | --- | --- |
| Arora 2020 ^46^ | Treatment | 12 of 20 RCTs | Canada (1), Israel (1), Norway (1), Poland (4), Spain (1), USA (4) | Cochrane Wounds, CENTRAL, MEDLINE, EMBASE, CINAHL | Inception to July 2019 | Electrical stimulation | N = 566, 50% male,  age 63-83 years,  mean 4 days to 12 months with pressure ulcer, Stage II (37%), III (45%),  coccygeal region (30%)  ischium (24%),  lower extremities including heels (23%)  greater trochanter of the femur (7%) | Surface area of pressure ulcers, time to complete healing, adverse events | The University of Sydney, Royal North Shore Hospital, NIHR |
| Ferris 2019 ^8^ | Prevalence, risk factors | 12 of 12 NRS | Japan (1), UK (2), US (2), Canada (1), Sweden (2), Italy (1), India (1), Poland (1) | Cochrane Wounds, CENTRAL, MEDLINE, EMBASE, CINAHL | Inception to Sept 2017 | NA | N = 63907, age 72 yrs,  stage I (41.31%), II (40,46%), III (12.82%), IV (2.56%),  Sacrum was the commonest site (range 38%–78.4%), followed by buttock, hip and heel | prevalence of pressure ulcers on admission to PCU, risk of pressure ulcers | No funding |
| Gillespie 2020 ^37^ | Prevention | 3 of 8 RCTs | US (1), Ireland (1), Belgium (1) | Cochrane Wounds, CENTRAL, MEDLINE, EMBASE, CINAHL | Inception to Feb 2019 | Repositioning regiments | N = 2018, age 85.1 yrs | Incidence of pressure ulcer, quality of life, adverse events | Griffith University, Qatar University, Norwich Medical School, Gold Coast Australia, NIHR |
| Hao 2017 ^47^ | Treatment | 1 of 3 RCT | US (1) | Cochrane Wounds, CENTRAL, MEDLINE, EMBASE, CINAHL | Inception to Sept 2016 | Topical phenytoin | N = 39, age 75.5 yrs,  Stage II decubitus ulcer | Time to complete healing, adverse events | NIHR |
| Joyce 2018 ^48^ | Prevention | 2 of 4 RCTs | Australia (1), Canada (1) | Cochrane Wounds, CENTRAL, MEDLINE, EMBASE, CINAHL | Inception to April 2018 | Healthcare service organization | N = 223, age 81-83 yrs,  30% male,  ≥ Stage II leg ulcers | Incidence of pressure ulcer, time to complete healing, quality of life, adverse events | Royal College of Surgeons in Ireland, University of Manchester, NIHR |
| Junkin 2009 ^38^ | Prevention | 1 of 1 NRS | Belgium (1) | MEDLINE, CINAHL | Inception to July 2009 | pressure redistribution surfaces or heel protection devices | N = 235, heel ulcers | Incidence of pressure ulcer | Not reported |
| Langer 2024 ^32(p20)^ | prevention, treatment | 26 of 33 RCTs | Italy (9), France (2), Spain (1), Japan (3), Israel (1), China (1), South Korea (1), Australia (3), USA (3), cross-continental (1), unclear (1) | Cochrane Wounds, CENTRAL, MEDLINE, EMBASE, CINAHL | Inception to May 2022 | Nutritional interventions | N = 2806, age ≥ 80yrs, 12.4%-67.5% male, Stage II: 15.4% to 100%, III: 14.1% to 50%, IV: 6.3% to 50%,  Sacrum: 16.6% to 64.3%, ankle: 16.6% to 20%, foot: 12.5% to 25% | Incidence of pressure ulcer, time to complete healing, rate of pressure ulcer healing, change in pressure ulcer area, adverse effects | NIHR, author institutions |
| Mäki‐Turja‐Rostedt 2019 ^50^ | Prevention | 10 RCT and 3 NRSI | United States (4), Netherlands (3), Canada (2), Italy (1), Belgium (1), Republic of Ireland (1), Norway (1), France (1), China (Hong Kong) (1), UK (1), unclear (1) | Cochrane Wounds, CENTRAL,  Scopus, MEDLINE,  Web of Science, CINAHL | Jan 2005 to Feb 2017 | Support surfaces, repositioning strategies, nutritional interventions | N = 21 to 94,789, age 73.2-92.5 yrs, 12.4%-67.5% male, Stage II: 15.4%-100%, Stage III: 14.1%-50%, Stage IV: 6.3%-50%, Sacrum: 16.6%-64.3%, Heel/ankle: 16.6%-20%, Foot: 12.5%-25% | Incidence of pressure ulcer, rate of pressure ulcer healing | Finland Government research funding (Satakunta Hospital District), Turku University Hospital |
| McInnes 2015 ^39^ | Prevention | 10 of 53 RCTs | Canada (2), USA (4), UK (1), Italy (1), Netherlands (1), unclear (1) | Cochrane Wounds, CENTRAL, MEDLINE, EMBASE, CINAHL | Inception to April 2015 | Supporting surfaces | N = 2799, age 55-82 yrs,  18%-50% male,  Stage 0 - I | Incidence of new pressure ulcers, change in pressure ulcer surface area | NIHR, University of York, University of Manchester |
| Moore 2018 ^40^ | Prevention | 7 of 18 RCTs | Spain (3), UK (3), Japan (1), Netherlands (1) | Cochrane Wounds, CENTRAL, MEDLINE, EMBASE, CINAHL | Inception to March 2017 | Wound dressing or skin topical agents | N = 1942, age 80-86.4 yrs, 19%-57% male | Pressure ulcer incidence, Time to ulcer development, quality of life, adverse events | NIHR, Royal College of Surgeons in Ireland |
| Porter-Armstrong 2018 ^41^ | Prevention | 1 of 5 RCT | Uk (1) | Cochrane Wounds, CENTRAL, MEDLINE, EMBASE, CINAHL | Inception to June 2017 | Healthcare educational programmes | Not reported | Pressure ulcer incidence, quality of life | NIHR, HSC Research and Development Division of the Public Health Agency |
| Shi 2021 ^42^ | Prevention | 7 of 32 RCTs | UK (8), France (1) | Cochrane Wounds, CENTRAL, MEDLINE, EMBASE, CINAHL | Inception to Nov 2019 | Alternating pressure (active) air beds or mattresses | N = 3574, age 69.05-87 yrs, 18%-50% male, No ulcers to stage 4 | Pressure ulcer incidence, time to pressure ulcer development, quality of life, adverse events | University of Manchester, NIHR |
| Shi 2021a ^43^ | Prevention | 4 of 17 RCTs | Belgium (1), USA (1), Netherlands (2) | Cochrane Wounds, CENTRAL, MEDLINE, EMBASE, CINAHL | Inception to Nov 2019 | Reactive air surfaces | N = 447, age 80-87 yrs, 19%-78% male | Pressure ulcer incidence, quality of life, adverse events | University of Manchester, NIHR |
| Shi 2021b ^44^ | Prevention | 4 of 29 RCTs | USA (1), France (1), Netherlands (2) | Cochrane Wounds, CENTRAL, MEDLINE, EMBASE, CINAHL | Inception to Nov 2019 | Foam surfaces | N = 276, age 68.8-85.1 yrs, 19%-78% male, Stage III or IV ulcer | Pressure ulcer incidence, quality of life, adverse events | University of Manchester, NIHR |
| Sugathapala 2023 ^51^ | Prevalence | 43 NRS | Europe (24), North America (11), Asia: (6), Oceania (4), South America (2) | MEDLINE, PubMed (NCBI), EMBASE, Cochrane Library, CINAHL, ProQuest Nursing and Allied Health Database | Jan 2000 to May 2022 | NA | N = 45 to 90,580, age 68.8-90+ yrs, Male = 19%-50.2%, Stage I–IV (most studies) | prevalence of pressure ulcers on admission to PCU | Griffith University Postgraduate Research Scholarship and Griffith University International Postgraduate Research Scholarship |
| Walker 2017 ^49^ | Treatment | 9 of 9 RCTs | UK (4), US (2), Greece (1), Poland (1), European countries (1) | Cochrane Wounds, CENTRAL, MEDLINE, EMBASE, CINAHL | Inception to Feb 2017 | Wound dressing | N = 483, age 58.5-83.8 yrs, 5%-65% male, mean 2 weeks to over 3 months with pressure ulcers,  Stage II: 12.82%-75%, Stage III: 25%-87.18%  Sacrum: 16.16% to 52.9%  Trochanter: 3.3% to 26.32%  Heel: 17.89% to 48.48%  Buttocks: 7.69% to 38.89%  Foot/Ankle: 5.9% to 17.95%  Leg: 2.78% to 13.89%  Ischium: 5.13% to 8.33% | Pressure ulcer incidence, time to complete healing, quality of life, adverse events | NHMRC, Griffith University, NHIR |
| Westby 2017 ^45^ | Treatment | 4 of 51 RCTs | Not reported | Cochrane Wounds, CENTRAL, MEDLINE, EMBASE, CINAHL | Inception to July 2016 | Wound dressing | N = 185, age 74-86 yrs,  mean 3.5 weeks to 6.5 weeks with pressure ulcers,  Stage II 77% to 100%, Stage III 0-100% | Time to complete healing | University of Manchester, NIHR |

## Appendix 6. Methodological quality of the reviews assessed by AMSTAR 2.0 tool.

| Review | research questions and inclusion criteria | Preplanned research methods | Explain study design selection | comprehensive literature search strategy | Study selection in duplicate | Data extraction in duplicate | Provided a list of excluded studies with justification | Describe the included studies in detail | Assessed Risk of Bias (RoB) in primary studies | Reported funding source | Used appropriate methods for combining data | assess the potential impact of RoB on results | Investigated publication bias | Reported potential sources of conflict of interest |
| --- | --- | --- | --- | --- | --- | --- | --- | --- | --- | --- | --- | --- | --- | --- |
| Arora 2020 (1) | Yes | Yes | Yes | Yes | Yes | Yes | Yes | Yes | Yes | Yes | Yes | Yes | Yes | Yes |
| Ferris 2019 (2) | Yes | Yes | Partial yes | Yes | Yes | No | Yes | No | No | NA | NA | No | No | NA |
| Gillespie 2020 | Yes | Yes | Yes | Yes | Yes | Yes | Yes | Yes | Yes | Yes | Yes | Yes | Yes | Yes |
| Hao 2017 (3) | Yes | Yes | Yes | Yes | Yes | Yes | Yes | Yes | Yes | Yes | Yes | Yes | Yes | Yes |
| Joyce 2018 (4) | Yes | Yes | Yes | Yes | Yes | Yes | Yes | Yes | Yes | Yes | Yes | Yes | Yes | Yes |
| Junkin 2009 (5) | No | No | Partial yes | No | No | No | No | No | No | NA | NA | No | No | NA |
| Langer 2024 (6) | Yes | Yes | Yes | Yes | Yes | Yes | Yes | Yes | Yes | Yes | Yes | Yes | Yes | Yes |
| Mäki-Turja-Rostedt 2019 (7) | Yes | No | Partial yes | Yes | Yes | Yes | No | Yes | Yes | NA | NA | No | No | No |
| McInnes 2015 (8) | Yes | Yes | Yes | Yes | Yes | Yes | Yes | Yes | Yes | Yes | Yes | Yes | Yes | Yes |
| Moore 2018 (9) | Yes | Yes | Yes | Yes | Yes | Yes | Yes | Yes | Yes | Yes | Yes | Yes | Yes | Yes |
| Porter-Armstrong 2018 (10) | Yes | Yes | Yes | Yes | Yes | Yes | Yes | Yes | Yes | Yes | Yes | Yes | Yes | Yes |
| Shi 2021 (11) | Yes | Yes | Yes | Yes | Yes | Yes | Yes | Yes | Yes | Yes | Yes | Yes | Yes | Yes |
| Shi 2021a (12) | Yes | Yes | Yes | Yes | Yes | Yes | Yes | Yes | Yes | Yes | Yes | Yes | Yes | Yes |
| Shi 2021b (13) | Yes | Yes | Yes | Yes | Yes | Yes | Yes | Yes | Yes | Yes | Yes | Yes | Yes | Yes |
| Sugathapala 2023 (14) | Yes | Yes | Partial yes | Yes | Yes | Yes | Yes | Yes | Yes | No | Yes | Yes | Yes | No |
| Walker 2017 (15) | Yes | Yes | Yes | Yes | Yes | Yes | Yes | Yes | Yes | Yes | Yes | Yes | Yes | Yes |
| Westby 2017 (16) | Yes | Yes | Yes | Yes | Yes | Yes | Yes | Yes | Yes | Yes | Yes | Yes | Yes | Yes |

## Appendix 7. Treatment and prevention of pressure injuries organized by outcome.

| Review ID | Intervention/comparison | Setting(s) | Results: treatment effect (95% C.I) | Number of participants (studies) | Certainty of evidence (GRADE) | Comments: Review authors’ assessment of the certainty of evidence |
| --- | --- | --- | --- | --- | --- | --- |
| Rate of pressure ulcer healing | | | | | | |
| Langer 2024 (6) | Protein, arginine, zinc and antioxidants vs  standard diet or placebo | hospital (1)  Hospital and long-term care settings (2) | Mean Difference (MD) using PUSH score: −2.71 [−4.82, −0.61]; I^2^ =  42% | 80 (3) | Very low certainty | NR |
|  | Arginine and micronutrients vs standard diet or  Placebo | hospital | Mean Difference (MD) using PUSH score: −0.48 [−3.80, 2.84]  DESIGN-R score: −1.60 [−9.53, 6.33] | 50 (1)^‖^  44 (1) | Very low certainty | NR |
|  | Protein vs standard diet | hospital | Mean Difference (MD) using PUSH score: −1.00 [−2.76, 0.76] | 16 (1) ^‖^ | Very low certainty | NR |
|  | Collagen vs standard diet or placebo | Long-term care settings (1), hospital (1)  hospital (1) | Mean Difference (MD) using PUSH score: −1.00 [−3.13, 1.14]; I^2^ = 74%  Mean Difference (MD) using DESIGN-R score: −6.00 [−10.76, −1.24] | 183 (2)  44 (1) | Very low certainty | NR |
| Mäki‐Turja‐Rostedt 2019 (7) | Wound care support team vs usual care | Long-term care settings | No significant improvement in pressure ulcer healing rates | 181 (1) | Not assessed | NR |
| Time to pressure ulcer development | | | | | | |
| Shi 2021 (11) | Alternating pressure (active) air surfaces versus  reactive air surfaces | Nursing home | Hazard Ratio (HR): 2.25 [1.05, 4.83] | 308 (1) | Low certainty | downgraded  twice for high risk of detection bias |
|  | Alternating pressure mattress vs Alternating pressure overlay | Geriatric care | Hazard Ratio (HR): 0.96 [0.73, 1.26] | 989 (1) | Not assessed | NR |
| Incidence of pressure ulcers (i.e., new pressure ulcers) | | | | | | |
| Gillespie 2020 (17) | repositioning every 2hrs vs 3hrs | Long-term care settings | Risk Ratio (RR): Range 0.9 to 4.6 | 798 (2) | Very low to low certainty | downgraded due to serious limitations in design and imprecision (low number of events and wide confidence intervals) |
|  | repositioning every 2hrs vs 4hrs | Long-term care settings | Risk Ratio (RR): 1.23 [0.89, 1.69] | 766 (2) | Very low certainty | Downgraded twice for lack of blinding of nursing staff and missing data, and once for imprecision (low number of events) |
|  | repositioning every 3hrs vs 4hrs | Long-term care settings | Risk Ratio (RR): 0.20 [0.04, 0.92] | 632 (1) | Low certainty | Downgraded twice: once due to risk of performance bias (lack of blinding of personnel) and once for imprecision (low number of events and wide confidence intervals) |
|  | repositioning every 4hrs vs 6hrs | Long-term care settings | Risk Ratio (RR): 0.73, [0.53, 1.02] | 132 (1) | Very low certainty | Downgraded three times due to serious limitations in design (lack of blinding of outcome assessors and personnel and missing outcome data) and imprecision |
|  | 30° tilt 3-hourly vs 6-hourly 90° tilt (overnight) | Long-term care settings | Risk Ratio (RR): 0.27, [0.08, 0.91] | 213 (1) | Very low certainty | Downgraded twice for lack of blinding of nursing staff and missing data, and twice for very serious imprecision (low number of events and wide confidence intervals) |
| Joyce 2018 (4) | Enhanced multidisciplinary teams (EMDTs) vs  usual care | Long-term care settings | Hazard Ratio (HR): 1.12 [0.74, 1.68] | 181 (1) | Very low certainty | Downgraded twice for very serious study limitations and twice for very serious imprecision |
| Junkin 2009 (5) | wedge-shaped viscoelastic foam cushion vs standard foam pillow. | Long-term care settings | Wedge-shaped viscoelastic foam cushion was more effective for the prevention of heel PU than were standard foam pillows^†^ | 235 (1) | Not assessed | NA |
| Langer 2024 (6) | Energy, protein and micronutrients vs standard diet | Hospital (2)  Long-term care settings (1) | Risk Ratio (RR): 0.92, [0.71, 1.19]; P = 0.52, I^2^ = 35% | 1634 (3) | Low certainty | Downgraded the  evidence by two levels to low certainty due to very serious risk of  bias |
|  | Protein supplements vs standard diet | Hospital | Risk Ratio (RR): 0.75 [0.49 to 1.14]; P = 0.18, I^2^ = 17% | 4264 (4) | Low certainty | Downgraded the evidence  by one level due to serious risk of bias (we rated three out of four  studies as some concerns) and by one level for imprecision |
|  | Disease-specific diet vs a standard diet | Long-term care settings | Risk Ratio (RR): 0.80 [0.36 to 1.75] | 34 (1) | Very low certainty | Downgraded the evidence by one level due to serious risk of  bias (we rated risk of bias as some concerns) and by two levels due  to very serious imprecision |
| Mäki‐Turja‐Rostedt 2019 (7) | Advanced wheelchair cushions vs standard foam cushions | Long-term care settings | Eight (6.7%) participants in the control group and one (0.9%) in the intervention group developed PUs (p = 0.04). | 180 (1) | Not assessed | NR |
|  | 30° tilt repositioning every 3 hrs vs 6-hr 90° rotation | Long-term care settings | Incidence Rate Ratio (RR): 0.27, [0.08, 0.93] | 197 (1) | Not assessed | NR |
|  | Computerised decision support system vs usual care | Long-term care settings | 12% vs 36%, p = 0.001 | 133 (1) | Not assessed | NR |
|  | Health IT-driven workflow redesign vs historical control | Long-term care settings | IRR = 0.409, p < 0.035 | 6,161 (1) | Not assessed | NR |
|  | Static air overlay mattress vs viscoelastic foam mattress | Long-term care settings | Fewer grade 2 - 4 PUs; no p-value reported | 74 (1) | Not assessed | NR |
|  | Air overlay mattress vs viscoelastic mattress | Long-term care settings | PU incidence lower in intervention group; no p-value reported | 39 (1) | Not assessed | NR |
|  | PU prevention protocol + repositioning vs standard care | Long-term care settings | PU development reduced; no significance reported | 235 (1) | Not assessed | NR |
|  | PU prevention bundle (education + skin care) vs baseline | Long-term care settings | 2.5% to 0.8%; no p-value reported | 124 (1) | Not assessed | NR |
|  | Foam mattress + air overlay vs foam mattress | Long-term care settings | No significant difference in PU incidence | 50 (1) | Not assessed | NR |
| McInnes 2015 (8) | foam and fibre mattress replacement (Maxifloat) vs 4-inch-thick foam overlay (Iris 3000) | Long-term care settings | Risk Ratio (RR): 0.42 [0.18, 0.96] | 40 (1) | Not assessed | Trial did not state the methods used for allocation concealment nor blinded outcome assessment clearly |
|  | combination of standard cold foam mattress and static air overlay vs standard cold foam mattress | Nursing home | Risk Ratio (RR): 3.59 [0.79, 16.25] | 83 (1) | Not assessed | NR |
|  | Viscoelastic (Akton)  mattress overlay vs 3D anti-decubitus (Airatext) mattress overlay | Long-term care settings | No patient developed a pressure ulcer | 50 (1) | Not assessed | Sample size was small (25 in each group) and the study may have  been at risk of Type 2 error |
|  | Gel mattress vs air-filled overlay | Nursing home | Risk Ratio (RR): 0.80 [0.24, 2.72] | 52 (1) | Not assessed | NR |
|  | pressure-reducing wheelchair seat cushion vs standard foam (eggcrate) cushion | Nursing home | Risk Ratio (RR): 0.68 [0.33, 1.42] | 32 (1) | Not assessed | NR |
|  | skin protection seat cushions vs segmented foam cushion | Nursing home | Risk Ratio (RR): 0.60 [0.31, 1.17] | 322 (1) | Not assessed | NR |
|  | Alternating Pressure overlay vs an Alternating Pressure mattress | Hospitals | Risk Ratio (RR): 1.04 [0.81, 1.35] | 1971 (1) | Not assessed | NR |
|  | Pegasus Airwave mattress vs Cairwave Therapy system | Unknown | No patients developed an ulcer within the 20-day follow- up in either arm of this trial. | 75 (1) | Not assessed | NR |
|  | gel pad (Jay gel) plus a foam wheelchair cushion vs foam cushion alone | Long-term care settings | Risk Ratio (RR): 0.61 [0.37, 1.00] | 141 (1) | Not assessed | NR |
|  | Slab foam seat cushion vs Bespoke contoured foam cushion | Long-term care settings | Risk Ratio (RR): 1.00 [0.81, 1.18] | 248 (1) | Not assessed | NR |
| Moore 2018 (9) | Topical application of fatty acid vs olive oil | Home care (1), nursing home (1) | Risk Ratio (RR): 1.28 [0.76, 2.17]; I^2^ = 0% | 1078 (2) | Low certainty | Downgraded twice for very serious imprecision due to  the small number of events and a wide CI |
|  | Topical application of fatty acid vs standard care | high-dependency unit at a university  hospital | Risk Ratio (RR): 0.53 [0.27, 1.04] | 171 (1) | Low certainty | downgraded once for serious risk of bias due  to performance bias and downgraded once for serious imprecision |
|  | Active lotion vs placebo | Geriatric care | Risk Ratio (RR): 0.73 [0.45, 1.19] | 319 (1) | Low certainty | Downgraded once for serious risk  of bias due to unclear risk of selection bias and downgraded once  for serious imprecision due to a wide CI |
|  | DSMO cream vs placebo | Nursing home | Risk Ratio (RR): 1.99 [1.1, 3.57] | 79 (1) | Very low certainty | Downgraded twice for very serious risk of  bias due to sequence generation, sequence allocation, and baseline  imbalance; and once for serious imprecision due to wide CIs |
|  | Conotrane versus placebo | Geriatric care | Risk Ratio (RR): 0.74 [0.52, 1.07] | 258 (1) | Very low certainty | Downgraded twice for very serious risk of  bias due to sequence generation, sequence allocation, and baseline  imbalance; and once for serious imprecision due to wide CIs |
| Porter-Armstrong 2018 (10) | Training, monitoring and observation vs  monitoring and observation | Residential homes | Risk Ratio (RR): 0.63 [0.37, 1.05] | 345 (1) | Very low certainty | Downgraded  twice for serious limitations due to performance bias, detection  bias and outcome reporting bias, and downgraded once for  imprecision due to a wide confidence interval |
|  | Training, monitoring and  observation vs observation alone | Residential homes | Risk Ratio (RR): 1.21 [0.60, 2.43] | 325 (1) | Very low certainty | Downgraded  twice for serious limitations due to performance bias, detection  bias and outcome reporting bias, and downgraded once for  imprecision due to a wide confidence interval |
|  | monitoring and observation  vs observation alone | Residential homes | Risk Ratio (RR): 1.93 [0.96, 3.88] | 232 (1) | Very low certainty | Downgraded  twice for serious limitations due to performance bias, detection  bias and outcome reporting bias, and downgraded once for  imprecision due to a wide confidence interval |
| Shi 2021 (11) | Alternating pressure (active) air surfaces vs  foam surfaces | community inpatient facilities (1), Long-term care settings and nursing homes (2) | Risk Ratio (RR): 0.79 [0.60, 1.03]; I^2^ = 0% | 2171 (3) | Low certainty | Evidence certainty was downgraded once for  risk of bias (two studies contributing 50% weight in the meta-  analysis had either one domain other than performance bias at  high risk of bias, or all domains at unclear risk of bias; two studies  contributing 50% of weight in the meta-analysis had domains other than performance bias at low or unclear risk of bias), and once for  imprecision (wide confidence interval crossed RR = 0.75) |
|  | Alternating pressure (active) air surfaces versus  reactive air surfaces | Nursing home | Risk Ratio (RR): 2.25 [1.01, 5.02] | 308 (1) | Not assessed | NA |
| Walker 2017 (15) | hydropolymer foam dressing vs silicone foam dressing | Geriatric care | Risk Ratio (RR): 0.89 [0.45, 1.75] | 38 (1) | Very low certainty | Downgraded once due to serious limitations in design and implementation (lack of blinding) and twice for very serious imprecision |
|  | Hydrocellular, hydropolymer and polyurethane foam dressings vs hydrocolloid dressings | Aged care and palliative settings (1), Community-dwelling (2) | Risk Ratio (RR): 0.85 [0.54, 1.34]; I^2^ = 5.73% | 198 (3) | Very low certainty | Downgraded once due to serious limitations in design and implementation (lack of blinding) and twice for very serious imprecision |
|  | polyurethane foam dressing vs hydrogel dressing | Palliative care unit | Risk Ratio (RR): 1.00 [0.78, 1.28] | 34 (1) | Very low certainty | Downgraded once due to limitations in design and implementation (lack of blinding) and twice for very serious imprecision |
|  | Polyurethane, silver and ibuprofen-releasing foam dressing vs gauze, saline-soaked gauze, low-adherence dressing secured by a vapour-permeable film | Aged care and palliative care settings | Risk Ratio (RR): 1.33 [0.62, 2.88] | 36 (1) | Very low certainty | Downgraded once because of limitations in design and implementation (lack of blinding) and twice for serious imprecision |
| Time to complete healing | | | | | | |
| Arora 2020 (1) | Electrical stimulation vs placebo electrical stimulation | Rehab and geriatric care | Hazard Ratio (HR): 1.05 [0.44, 2.51] | 38 (1)^‡^ | Not assessed | NA |
| Hao 2017 (3) | Topical phenytoin vs hydrocolloid dressing | Long-term care settings | phenytoin group was 35.3 ± 14.3 days (mean ± standard deviation (SD)) days compared with 51.8 ± 19.6 (mean ± SD) days for the hydrocolloid dressing (Duoderm) group | 28 (1) | Not assessed | Authors noted that due to uncertainty regarding the number of participants covered by the reported data, the results are not appropriate for use |
|  | Topical phenytoin vs triple antibiotic ointment | Long-term care settings | phenytoin group was 35.3 ±14.3 days (mean ± standard deviation (SD)) days compared with 53.8 ± 8.5 (mean ± SD) days for triple antibiotic ointment group | 26 (1) | Not assessed | Authors noted that due to uncertainty regarding the number of participants covered by the reported data, the results are not appropriate for use |
| Joyce 2018 (4) | Enhanced multidisciplinary teams (EMDTs) vs  usual care | Long-term care settings | Hazard Ratio (HR): 1.48 [0.79, 2.78] | 181 (1) | Very low certainty | Downgraded twice for very serious study limitations and  twice for very serious imprecision |
| Joyce 2018 (4) | Multidisciplinary wound care vs  usual care | Long-term care settings | Hazard Ratio (HR): 1.73 [1.20, 2.50] | 176 (1) | Very low certainty | Downgraded twice for very serious study limitations and  twice for very serious imprecision |
| Langer 2024 (6) | EPA, GLA and antioxidants vs standard diet | Unknown | Risk Ratio (RR): 9.00 [0.59, 137.65] {pressure ulcers healed} | 24 (1) | Very low certainty | Downgraded the evidence by one level because of serious risk of bias (we rated risk of bias as some concerns) and by two levels due  to very serious imprecision |
| Walker 2017 (15) | polyurethane foam dressing vs hydrogel dressing | Palliative care unit | Compared to the hydrogel dressings, foam dressings were associated with an increased number of treatment days MD: 5.67 days, (95% CI -4.03 to 15.37) | 34 (1) | Very low certainty | Downgraded once due to limitations in design and implementation (lack of blinding) and twice for very serious imprecision |
|  | Polyurethane, silver and ibuprofen-releasing foam dressing vs gauze, saline-soaked gauze, low-adherence dressing secured by a vapour-permeable film | Community dwelling | foam dressings were associated with a decreased time to complete healing MD -35.8 days, (95% CI -56.77 to -14.83) | 95 (1) | Very low certainty | Downgraded once because of limitations in design and implementation (lack of blinding) and twice for serious imprecision |
| Westby 2017 (16) | Advanced dressing vs basic dressing | Long-term care settings | Hazard Ratio (HR): 1.13 [0.42, 3.00] | 36 (1) | Not assessed | NA |
|  | Protease-modulating dressing vs advanced dressing | Long-term care settings | Hazard Ratio (HR): 1.34 [0.67, 2.65] | 65 (1) | Not assessed | NA |
|  | Hydrocolloid +/- alginate vs ineligible: radiant heat | Long-term care settings | Hazard Ratio (HR): 0.64[0.23, 1.77] | 41 (1) | Not assessed | NA |
|  | Hydrogel vs hydrocolloid | Long-term care settings | Hazard Ratio (HR): 1.75 [1.00, 3.05] | 43 (1) | Not assessed | NA |
| Adverse events | | | | | | |
| Arora 2020 (1) | Electrical stimulation vs placebo or sham electrical stimulation | rehab and geriatric care (5), medical centres (2), residential care centre (2), community-based centre (1) | In 1 trial, 15% reporting minor tingling, experienced comorbidity-related events (4/10 trials), and no events (5/10 trials) | 479 (10) | Low certainty | Downgraded once for serious risk of bias (several studies had either high or unclear risk of bias for selection and attrition bias) and once for imprecision |
| Gillespie 2020 (17) | repositioning regimens vs other standard practices or with alternative repositioning regimens | Any setting | No studies found | NA | NA | NA |
| Hao 2017 (3) | Topical phenytoin vs placebo/alternative treatments/no therapy | Any setting | No studies found | NA | NA | NA |
| Joyce 2018 (4) | Enhanced multidisciplinary teams (EMDTs) vs  usual care | Long-term care settings | Hospitalization Hazard Ratio (HR): 1.20 [0.62, 2.36]  Emergency department visits HR: 1.30 [0.58, 2.90] | 181 (1) | Very low certainty | Downgraded twice for very serious study limitations and  twice for very serious imprecision |
| Langer 2024 (6) | Protein supplements vs standard diet | hospital | Gastrointestinal side effects (unspecified) Risk Ratio (RR): 0.70 [0.06, 7.96]; I^2^ =  65% | 140 (2) | Very low certainty | Downgraded the evidence by  one level due to serious risk of bias (we rated both studies as some  concerns) and by two levels for very serious imprecision |
|  | Protein, arginine, zinc and antioxidants vs standard diet or placebo | Long-term care settings | Gastrointestinal side effects (diarrhea, nausea, vomiting, constipation, and dyspepsia) Risk Ratio (RR): 1.17 [0.77, 1.79] | 43 (1) | Very low certainty | Downgraded the certainty of evidence by one level for serious  risk of bias (we rated risk of bias as some concerns) and by two  levels for very serious imprecision |
|  | Arginine and micronutrients vs standard diet or  placebo | hospital (2), Long-term care settings (1) | Gastrointestinal side effects (diarrhea, nausea) Risk Ratio (RR): 1.54 [0.36, 6.64]; I^2^ = 9% | 282 (3) | Very low certainty | downgraded the evidence by one level due to serious risk of bias (we rated one study at high risk of bias) and twice for very serious  imprecision |
|  | Protein vs standard diet | Long-term care settings | Gastrointestinal side effects (unspecified) Risk Ratio (RR): 0.15 [0.02, 1.22] | 152 (1) | Very low certainty | Downgraded the evidence by one level due  to serious risk of bias (we rated the study as high risk of bias) and  by two levels for very serious imprecision |
|  | Ornithine alpha-ketoglutarate vs with placebo | Geriatric care | Gastrointestinal side effects (unspecified) Risk Ratio (RR): 1.10 [0.55, 2.20] | 160 (1) | Very low certainty | Downgraded  the evidence by one level due to serious risk of bias (we rated risk  of bias as some concerns) and by two levels due to very serious  imprecision |
| Moore 2018 (9) | Topical application of fatty acid vs olive oil | Nursing home (1) | Adverse events (unspecified) Risk Ratio (RR): 2.22 [0.2, 24.37]  No studies reported length of hospital stay | 247 (1) | Low certainty | Downgraded twice for very  serious imprecision due to a very small number of events leading  to a very wide CI |
| Walker 2017 (15) | hydropolymer foam dressing vs silicone foam dressing | Geriatric care | Adverse events (unspecified) Risk Ratio (RR): 0.37 [0.04, 3.25] | 38 (1) | Very low certainty | Downgraded once due to serious limitations in design and implementation (lack of blinding) and twice for very serious imprecision |
|  | Hydrocellular, hydropolymer and polyurethane foam dressings vs hydrocolloid dressings | Aged care and palliative settings (1), Community-dwelling (2) | Adverse events (unspecified) Risk Ratio (RR): 0.88 [0.37, 2.11]; I^2^ = 0% | 198 (3) | Very low certainty | Downgraded once due to serious limitations in design and implementation (lack of blinding) and twice for very serious imprecision |
|  | Polyurethane, silver and ibuprofen-releasing foam dressing vs gauze, saline-soaked gauze, low-adherence dressing secured by a vapour-permeable film | Community dwelling | Risk Ratio (RR): 0.58 [0.33, 1.05] | 95 (1) | Very low certainty | Downgraded once because of limitations in design and implementation (lack of blinding) and twice for serious imprecision |
| Quality of life | | | | | | |
| Gillespie 2020 (17) | repositioning regimens vs other standard practices or with alternative repositioning regimens | Any setting | No studies found | NA | NA | NA |
| Joyce 2018 (4) | Enhanced multidisciplinary teams (EMDTs) vs  usual care | Long-term care settings | Mean utilities measured using Q5D: 0.03 [-0.03, 0.09] | 181 (1) | Very low certainty | Downgraded twice for very serious study limitations and  twice for very serious imprecision |
| Moore 2018 (9) | Fatty acid vs other topical applications | Any setting | No studies found | NA | NA | NA |
| Shi 2021 (11) | Alternating pressure (active) air surfaces vs  foam surfaces | Community inpatient facilities | Mean Difference (MD) using 90-day EQ-5D-5L: 0.00 [-0.05, 0.05]  Mean Difference (MD) using 90-day PU-QoL-UI: 0.00 [-0.03, 0.03] | 267 (1)  233 (1) | Low certainty | Downgraded twice for imprecision due to small sample sizes for this outcome |
| Walker 2017 (15) | polyurethane foam dressing vs hydrogel dressing | Palliative care unit | Risk Ratio (RR): 0.33 [0.01, 7.65] | 34 (1) | Very low certainty | Downgraded once due to limitations in design and implementation (lack of blinding) and twice for very serious imprecision |
| Change in pressure ulcer surface area (cm^2^) | | | | | | |
| Arora 2020 (1) | Electrical stimulation vs placebo or sham electrical stimulation | rehab and geriatric care (3), medical centres (2), residential care centre (2), community-based centre (1) | Mean Difference (MD): ranged from −0.90 cm^2^ to 5.55 cm^2§^ | 336 (8) | Very low certainty | Downgraded once for serious risk of bias (several studies had either high or unclear risk of bias for selection and attrition bias), once for imprecision and once for inconsistency |
| Langer 2024 (6) | Protein, arginine, zinc and antioxidants vs  standard diet or placebo | hospitals (1), long term-  care settings (1) | Mean Difference (MD): −2.00 cm^2^ [−4.54, 0.53]; I^2^ = 13% | 71 (2) | Very low certainty | Downgraded the certainty of evidence  by one level for serious risk of bias (we rated the risk of bias of  the study with more weight as some concerns) and by two levels  for very serious imprecision |
|  | Arginine and micronutrients vs standard diet or  placebo | hospitals (1), long term-  care settings (1) | Mean Difference (MD): −15.80 cm^2^ [−25.11, −6.48]; I^2^ = 0% | 231 (2) | Low certainty | Downgraded the  evidence by one level due to serious risk of bias (one of two studies  had a high overall risk of bias) and by one level due to serious  imprecision |
|  | Collagen vs standard diet or placebo | hospitals | Mean Difference (MD): −1.81 cm^2^ [−3.36, −0.26] | 74 (1) | Moderate certainty | Downgraded  the evidence by one level due to serious imprecision |
|  | Ornithine alpha-ketoglutarate vs placebo | Geriatric care | No differences in wound area changes PU > 8 cm^2^ (data not reported), PU ≤ 8 cm2: Mean Difference (MD): −0.60 [−1.90, 0.70] | 22 (1) | Very low certainty | Downgraded the evidence for both outcomes to very low certainty; by one level due to serious risk of bias (we rated risk of bias as some  concerns) and by two levels due to very serious imprecision |
| McInnes 2015 (8) | "Low-tech" CLP support surfaces vs "High-tech" support surfaces | Any setting | No studies found | NA | NA | NA |
| Walker 2017 (15) | hydropolymer foam dressing vs silicone foam dressing | Geriatric care | hydropolymer foam dressing had a mean reduction in would area of 3.3 cm^2^ compared to 3.1 cm^2^ silicone foam dressing | 38 (1) | Very low certainty | Downgraded once due to serious limitations in design and implementation (lack of blinding) and twice for very serious imprecision |
|  | polyurethane foam dressing vs hydrogel dressing | Palliative care unit | Mean difference (MD) reduction of ulcer size  was 0.30 cm^2^ per day (95% CI -0.15 to 0.75) | 30 (1) | Very low certainty | Downgraded once due to serious limitations in design and implementation (lack of blinding) and twice for very serious imprecision |

^*^Four studies with a total of 205 participants examined time to complete healing for relevant populations (Adunksy 2005; Asbjornsen 1990; Feeder 1991; Polak 2017) but only one (Adunksy 2005) provided sufficient information.

†Review authors noted that data was not sufficiently detailed or comparable to analyse quantitatively.

^‡^Kloth 1988 examined the surface area of pressure ulcers but did not report sufficient information

^§^Data was not pooled bec considerable statistical heterogeneity between studies (I^2^ = 96%)

^‖^Benati 2001 provided only graphically presented changes in PSST scores showing that participants who received protein supplements seemed to have a more rapid improvement in pressure ulcer healing over the 15-day intervention period compared with those who received a standard diet.

## Appendix 8. Categorization of treatment and prevention of pressure injuries by types of interventions.

| **Category** | **Review ID** | **Intervention/comparison** | **Outcome** | **Setting(s)** | **Results: treatment effect (95% C.I)** | **Number of participants (studies)** | **Certainty of evidence (GRADE)** |
| --- | --- | --- | --- | --- | --- | --- | --- |
| Electrical stimulation | Arora 2020 (1) | Electrical stimulation vs placebo electrical stimulation | Time to complete healing | Rehab and geriatric care | Hazard Ratio (HR): 1.05 [0.44, 2.51] | 38 (1)‡ | Not assessed |
|  |  | Electrical stimulation vs placebo or sham electrical stimulation | Adverse events | rehab and geriatric care (5), medical centres (2), residential care centre (2), community-based centre (1) | In 1 trial, 15% reporting minor tingling, experienced comorbidity-related events (4/10 trials), and no events (5/10 trials) | 479 (10) | Low certainty |
|  |  | Electrical stimulation vs placebo or sham electrical stimulation | Change in pressure ulcer surface area (cm2) | rehab and geriatric care (3), medical centres (2), residential care centre (2), community-based centre (1) | Mean Difference (MD): ranged from −0.90 cm2 to 5.55 cm2§ | 336 (8) | Very low certainty |
| Nutrition | Langer 2024 (6) | Protein, arginine, zinc and antioxidants vs  standard diet or placebo | Rate of pressure ulcer healing | hospital (1) Hospital and long-term care settings (2) | Mean Difference (MD) using PUSH score: −2.71 [−4.82, −0.61]; I2 = 42% | 80 (3) | Very low certainty |
|  |  | Arginine and micronutrients vs standard diet or Placebo | Rate of pressure ulcer healing | hospital | Mean Difference (MD) using PUSH score: −0.48 [−3.80, 2.84] DESIGN-R score: −1.60 [−9.53, 6.33] | 50 (1)‖   44 (1) | Very low certainty |
|  |  | Protein vs standard diet | Rate of pressure ulcer healing | hospital | Mean Difference (MD) using PUSH score: −1.00 [−2.76, 0.76] | 16 (1) ‖ | Very low certainty |
|  |  | Collagen vs standard diet or placebo | Rate of pressure ulcer healing | Long-term care settings (1), hospital (1)   hospital (1) | Mean Difference (MD) using PUSH score: −1.00 [−3.13, 1.14]; I2 = 74%  Mean Difference (MD) using DESIGN-R score: −6.00 [−10.76, −1.24] | 183 (2)    44 (1) | Very low certainty |
|  |  | Energy, protein and micronutrients vs standard diet | Incidence of pressure ulcers (i.e., new pressure ulcers) | Hospital (2) Long-term care settings (1) | Risk Ratio (RR): 0.92, [0.71, 1.19]; P = 0.52, I2 = 35% | 1634 (3) | Low certainty |
|  |  | Protein supplements vs standard diet | Incidence of pressure ulcers (i.e., new pressure ulcers) | Hospital | Risk Ratio (RR): 0.75 [0.49 to 1.14]; P = 0.18, I2 = 17% | 4264 (4) | Low certainty |
|  |  | Disease-specific diet vs a standard diet | Incidence of pressure ulcers (i.e., new pressure ulcers) | Long-term care settings | Risk Ratio (RR): 0.80 [0.36 to 1.75] | 34 (1) | Very low certainty |
|  |  | EPA, GLA and antioxidants vs standard diet | Time to complete healing | Unknown | Risk Ratio (RR): 9.00 [0.59, 137.65] {pressure ulcers healed} | 24 (1) | Very low certainty |
|  |  | Protein supplements vs standard diet | Adverse events | hospital | Gastrointestinal side effects (unspecified) Risk Ratio (RR): 0.70 [0.06, 7.96]; I2 = 65% | 140 (2) | Very low certainty |
|  |  | Protein, arginine, zinc and antioxidants vs standard diet or placebo | Adverse events | Long-term care settings | Gastrointestinal side effects (diarrhea, nausea, vomiting, constipation, and dyspepsia) Risk Ratio (RR): 1.17 [0.77, 1.79] | 43 (1) | Very low certainty |
|  |  | Arginine and micronutrients vs standard diet or placebo | Adverse events | hospital (2), Long-term care settings (1) | Gastrointestinal side effects (diarrhea, nausea) Risk Ratio (RR): 1.54 [0.36, 6.64]; I2 = 9% | 282 (3) | Very low certainty |
|  |  | Protein vs standard diet | Adverse events | Long-term care settings | Gastrointestinal side effects (unspecified) Risk Ratio (RR): 0.15 [0.02, 1.22] | 152 (1) | Very low certainty |
|  |  | Ornithine alpha-ketoglutarate vs with placebo | Adverse events | Geriatric care | Gastrointestinal side effects (unspecified) Risk Ratio (RR): 1.10 [0.55, 2.20] | 160 (1) | Very low certainty |
|  |  | Protein, arginine, zinc and antioxidants vs  standard diet or placebo | Change in pressure ulcer surface area (cm2) | hospitals (1), long term- care settings (1) | Mean Difference (MD): −2.00 cm2 [−4.54, 0.53]; I2 = 13% | 71 (2) | Very low certainty |
|  |  | Arginine and micronutrients vs standard diet or placebo | Change in pressure ulcer surface area (cm2) | hospitals (1), long term- care settings (1) | Mean Difference (MD): −15.80 cm2 [−25.11, −6.48]; I2 = 0% | 231 (2) | Low certainty |
|  |  | Collagen vs standard diet or placebo | Change in pressure ulcer surface area (cm2) | hospitals | Mean Difference (MD): −1.81 cm2 [−3.36, −0.26] | 74 (1) | Moderate certainty |
|  |  | Ornithine alpha-ketoglutarate vs placebo | Change in pressure ulcer surface area (cm2) | Geriatric care | No differences in wound area changes PU > 8 cm2 (data not reported), PU ≤ 8 cm2: Mean Difference (MD): −0.60 [−1.90, 0.70] | 22 (1) | Very low certainty |
| Organizational strategies | Joyce 2018 (4) | Enhanced multidisciplinary teams (EMDTs) vs usual care | Incidence of pressure ulcers (i.e., new pressure ulcers) | Long-term care settings | Hazard Ratio (HR): 1.12 [0.74, 1.68] | 181 (1) | Very low certainty |
|  |  | Enhanced multidisciplinary teams (EMDTs) vs usual care | Time to complete healing | Long-term care settings | Hazard Ratio (HR): 1.48 [0.79, 2.78] | 181 (1) | Very low certainty |
|  |  | Multidisciplinary wound care vs usual care | Time to complete healing | Long-term care settings | Hazard Ratio (HR): 1.73 [1.20, 2.50] | 176 (1) | Very low certainty |
|  |  | Enhanced multidisciplinary teams (EMDTs) vs usual care | Adverse events | Long-term care settings | Hospitalization Hazard Ratio (HR): 1.20 [0.62, 2.36] Emergency department visits HR: 1.30 [0.58, 2.90] | 181 (1) | Very low certainty |
|  |  | Enhanced multidisciplinary teams (EMDTs) vs usual care | Quality of life | Long-term care settings | Mean utilities measured using Q5D: 0.03 [-0.03, 0.09] | 181 (1) | Very low certainty |
|  | Porter-Armstrong 2018 (10) | Training, monitoring and observation vs monitoring and observation | Incidence of pressure ulcers (i.e., new pressure ulcers) | Residential homes | Risk Ratio (RR): 0.63 [0.37, 1.05] | 345 (1) | Very low certainty |
|  |  | monitoring and observation vs observation alone | Incidence of pressure ulcers (i.e., new pressure ulcers) | Residential homes | Risk Ratio (RR): 1.93 [0.96, 3.88] | 232 (1) | Very low certainty |
|  |  | Training, monitoring and observation vs observation alone | Incidence of pressure ulcers (i.e., new pressure ulcers) | Residential homes | Risk Ratio (RR): 1.21 [0.60, 2.43] | 325 (1) | Very low certainty |
|  | Mäki‐Turja‐Rostedt 2019 (7) | Wound care support team vs usual care | Rate of pressure ulcer healing | Long-term care settings | No significant improvement in pressure ulcer healing rates | 181 (1) | Not assessed |
|  |  | Computerised decision support system vs usual care | Incidence of pressure ulcers (i.e., new pressure ulcers) | Long-term care settings | 12% vs 36%, p = 0.001 | 133 (1) | Not assessed |
|  |  | Health IT-driven workflow redesign vs historical control | Incidence of pressure ulcers (i.e., new pressure ulcers) | Long-term care settings | IRR = 0.409, p < 0.035 | 6,161 (1) | Not assessed |
|  |  | PU prevention protocol + repositioning vs standard care | Incidence of pressure ulcers (i.e., new pressure ulcers) | Long-term care settings | PU development reduced; no significance reported | 235 (1) | Not assessed |
|  |  | PU prevention bundle (education + skin care) vs baseline | Incidence of pressure ulcers (i.e., new pressure ulcers) | Long-term care settings | 2.5% to 0.8%; no p-value reported | 124 (1) | Not assessed |
| Reposition strategies | Gillespie 2020 (17) | repositioning every 2hrs vs 3hrs | Incidence of pressure ulcers (i.e., new pressure ulcers) | Long-term care settings | Risk Ratio (RR): Range 0.9 to 4.6 | 798 (2) | Very low to low certainty |
|  |  | repositioning every 2hrs vs 4hrs | Incidence of pressure ulcers (i.e., new pressure ulcers) | Long-term care settings | Risk Ratio (RR): 1.23 [0.89, 1.69] | 766 (2) | Very low certainty |
|  |  | repositioning every 3hrs vs 4hrs | Incidence of pressure ulcers (i.e., new pressure ulcers) | Long-term care settings | Risk Ratio (RR): 0.20 [0.04, 0.92] | 632 (1) | Low certainty |
|  |  | repositioning every 4hrs vs 6hrs | Incidence of pressure ulcers (i.e., new pressure ulcers) | Long-term care settings | Risk Ratio (RR): 0.73, [0.53, 1.02] | 132 (1) | Very low certainty |
|  |  | 30° tilt 3-hourly vs 6-hourly 90° tilt (overnight) | Incidence of pressure ulcers (i.e., new pressure ulcers) | Long-term care settings | Risk Ratio (RR): 0.27, [0.08, 0.91] | 213 (1) | Very low certainty |
|  |  | repositioning regimens vs other standard practices or with alternative repositioning regimens | Adverse events | Any setting | No studies found | NA | NA |
|  |  | repositioning regimens vs other standard practices or with alternative repositioning regimens | Quality of life | Any setting | No studies found | NA | NA |
|  | Mäki‐Turja‐Rostedt 2019 (7) | 30° tilt repositioning every 3 hrs vs 6-hr 90° rotation | Incidence of pressure ulcers (i.e., new pressure ulcers) | Long-term care settings | Incidence Rate Ratio (RR): 0.27, [0.08, 0.93] | 197 (1) | Not assessed |
| Support surfaces | McInnes 2015 (8) | "Low-tech" CLP support surfaces vs "High-tech" support surfaces | Change in pressure ulcer surface area (cm2) | Any setting | No studies found | NA | NA |
|  |  | Alternating Pressure overlay vs an Alternating Pressure mattress | Incidence of pressure ulcers (i.e., new pressure ulcers) | Hospitals | Risk Ratio (RR): 1.04 [0.81, 1.35] | 1971 (1) | Not assessed |
|  |  | combination of standard cold foam mattress and static air overlay vs standard cold foam mattress | Incidence of pressure ulcers (i.e., new pressure ulcers) | Nursing home | Risk Ratio (RR): 3.59 [0.79, 16.25] | 83 (1) | Not assessed |
|  | Junkin 2009 (5) | wedge-shaped viscoelastic foam cushion vs standard foam pillow. | Incidence of pressure ulcers (i.e., new pressure ulcers) | Long-term care settings | Wedge-shaped viscoelastic foam cushion was more effective for the prevention of heel PU than were standard foam pillows† | 235 (1) | Not assessed |
|  | McInnes 2015 (8) | foam and fibre mattress replacement (Maxifloat) vs 4-inch-thick foam overlay (Iris 3000) | Incidence of pressure ulcers (i.e., new pressure ulcers) | Nursing home | Risk Ratio (RR): 0.42 [0.18, 0.96] | 40 (1) | Not assessed |
|  |  | Gel mattress vs air-filled overlay | Incidence of pressure ulcers (i.e., new pressure ulcers) | Nursing home | Risk Ratio (RR): 0.80 [0.24, 2.72] | 52 (1) | Not assessed |
|  |  | gel pad (Jay gel) plus a foam wheelchair cushion vs foam cushion alone | Incidence of pressure ulcers (i.e., new pressure ulcers) | Long-term care settings | Risk Ratio (RR): 0.61 [0.37, 1.00] | 141 (1) | Not assessed |
|  |  | Pegasus Airwave mattress vs Cairwave Therapy system | Incidence of pressure ulcers (i.e., new pressure ulcers) | Unknown | No patients developed an ulcer within the 20-day follow- up in either arm of this trial. | 75 (1) | Not assessed |
|  |  | pressure-reducing wheelchair seat cushion vs standard foam (eggcrate) cushion | Incidence of pressure ulcers (i.e., new pressure ulcers) | Nursing home | Risk Ratio (RR): 0.68 [0.33, 1.42] | 32 (1) | Not assessed |
|  |  | skin protection seat cushions vs segmented foam cushion | Incidence of pressure ulcers (i.e., new pressure ulcers) | Nursing home | Risk Ratio (RR): 0.60 [0.31, 1.17] | 322 (1) | Not assessed |
|  |  | Slab foam seat cushion vs Bespoke contoured foam cushion | Incidence of pressure ulcers (i.e., new pressure ulcers) | Long-term care settings | Risk Ratio (RR): 1.00 [0.81, 1.18] | 248 (1) | Not assessed |
|  |  | Viscoelastic (Akton) mattress overlay vs 3D anti-decubitus (Airatext) mattress overlay | Incidence of pressure ulcers (i.e., new pressure ulcers) | Long-term care settings | No patient developed a pressure ulcer | 50 (1) | Not assessed |
|  | Shi 2021 (11) | Alternating pressure (active) air surfaces versus reactive air surfaces | Incidence of pressure ulcers (i.e., new pressure ulcers) | Nursing home | Risk Ratio (RR): 2.25 [1.01, 5.02] | 308 (1) | Not assessed |
|  |  | Alternating pressure (active) air surfaces vs foam surfaces | Incidence of pressure ulcers (i.e., new pressure ulcers) | community inpatient facilities (1), Long-term care settings and nursing homes (2) | Risk Ratio (RR): 0.79 [0.60, 1.03]; I2 = 0% | 2171 (3) | Low certainty |
|  |  | Alternating pressure (active) air surfaces vs foam surfaces | Quality of life | Community inpatient facilities | Mean Difference (MD) using 90-day EQ-5D-5L: 0.00 [-0.05, 0.05]  Mean Difference (MD) using 90-day PU-QoL-UI: 0.00 [-0.03, 0.03] | 267 (1)    233 (1) | Low certainty |
|  |  | Alternating pressure (active) air surfaces versus reactive air surfaces | Time to pressure ulcer development | Nursing home | Hazard Ratio (HR): 2.25 [1.05, 4.83] | 308 (1) | Low certainty |
|  |  | Alternating pressure mattress vs Alternating pressure overlay | Time to pressure ulcer development | Geriatric care | Hazard Ratio (HR): 0.96 [0.73, 1.26] | 989 (1) | Not assessed |
|  | Mäki‐Turja‐Rostedt 2019 (7) | Advanced wheelchair cushions vs standard foam cushions | Incidence of pressure ulcers (i.e., new pressure ulcers) | Long-term care settings | Eight (6.7%) participants in the control group and one (0.9%) in the intervention group developed PUs (p = 0.04). | 180 (1) | Not assessed |
|  |  | Static air overlay mattress vs viscoelastic foam mattress | Incidence of pressure ulcers (i.e., new pressure ulcers) | Long-term care settings | Fewer grade 2 - 4 PUs; no p-value reported | 74 (1) | Not assessed |
|  |  | Air overlay mattress vs viscoelastic mattress | Incidence of pressure ulcers (i.e., new pressure ulcers) | Long-term care settings | PU incidence lower in intervention group; no p-value reported | 39 (1) | Not assessed |
|  |  | Foam mattress + air overlay vs foam mattress | Incidence of pressure ulcers (i.e., new pressure ulcers) | Long-term care settings | No significant difference in PU incidence | 50 (1) | Not assessed |
| Topical and dressing interventions | Walker 2017 (15) | Hydrocellular, hydropolymer and polyurethane foam dressings vs hydrocolloid dressings | Adverse events | Aged care and palliative settings (1), Community-dwelling (2) | Adverse events (unspecified) Risk Ratio (RR): 0.88 [0.37, 2.11]; I2 = 0% | 198 (3) | Very low certainty |
|  |  | hydropolymer foam dressing vs silicone foam dressing | Adverse events | Geriatric care | Adverse events (unspecified) Risk Ratio (RR): 0.37 [0.04, 3.25] | 38 (1) | Very low certainty |
|  |  | Polyurethane, silver and ibuprofen-releasing foam dressing vs gauze, saline-soaked gauze, low-adherence dressing secured by a vapour-permeable film | Adverse events | Community dwelling | Risk Ratio (RR): 0.58 [0.33, 1.05] | 95 (1) | Very low certainty |
|  | Moore 2018 (9) | Topical application of fatty acid vs olive oil | Adverse events | Nursing home (1) | Adverse events (unspecified) Risk Ratio (RR): 2.22 [0.2, 24.37]  No studies reported length of hospital stay | 247 (1) | Low certainty |
|  |  | Active lotion vs placebo | Incidence of pressure ulcers (i.e., new pressure ulcers) | Geriatric care | Risk Ratio (RR): 0.73 [0.45, 1.19] | 319 (1) | Low certainty |
|  |  | Conotrane versus placebo | Incidence of pressure ulcers (i.e., new pressure ulcers) | Geriatric care | Risk Ratio (RR): 0.74 [0.52, 1.07] | 258 (1) | Very low certainty |
|  |  | DSMO cream vs placebo | Incidence of pressure ulcers (i.e., new pressure ulcers) | Nursing home | Risk Ratio (RR): 1.99 [1.1, 3.57] | 79 (1) | Very low certainty |
|  |  | Topical application of fatty acid vs olive oil | Incidence of pressure ulcers (i.e., new pressure ulcers) | Home care (1), nursing home (1) | Risk Ratio (RR): 1.28 [0.76, 2.17]; I2 = 0% | 1078 (2) | Low certainty |
|  |  | Topical application of fatty acid vs standard care | Incidence of pressure ulcers (i.e., new pressure ulcers) | high-dependency unit at a university hospital | Risk Ratio (RR): 0.53 [0.27, 1.04] | 171 (1) | Low certainty |
|  |  | Fatty acid vs other topical applications | Quality of life | Any setting | No studies found | NA | NA |
|  | Walker 2017 (15) | hydropolymer foam dressing vs silicone foam dressing | Change in pressure ulcer surface area (cm2) | Geriatric care | hydropolymer foam dressing had a mean reduction in would area of 3.3 cm2 compared to 3.1 cm2 silicone foam dressing | 38 (1) | Very low certainty |
|  |  | Hydrocellular, hydropolymer and polyurethane foam dressings vs hydrocolloid dressings | Incidence of pressure ulcers (i.e., new pressure ulcers) | Aged care and palliative settings (1), Community-dwelling (2) | Risk Ratio (RR): 0.85 [0.54, 1.34]; I2 = 5.73% | 198 (3) | Very low certainty |
|  |  | polyurethane foam dressing vs hydrogel dressing | Change in pressure ulcer surface area (cm2) | Palliative care unit | Mean difference (MD) reduction of ulcer size  was 0.30 cm2 per day (95% CI -0.15 to 0.75) | 30 (1) | Very low certainty |
|  |  | hydropolymer foam dressing vs silicone foam dressing | Incidence of pressure ulcers (i.e., new pressure ulcers) | Geriatric care | Risk Ratio (RR): 0.89 [0.45, 1.75] | 38 (1) | Very low certainty |
|  |  | polyurethane foam dressing vs hydrogel dressing | Incidence of pressure ulcers (i.e., new pressure ulcers) | Palliative care unit | Risk Ratio (RR): 1.00 [0.78, 1.28] | 34 (1) | Very low certainty |
|  |  | Polyurethane, silver and ibuprofen-releasing foam dressing vs gauze, saline-soaked gauze, low-adherence dressing secured by a vapour-permeable film | Incidence of pressure ulcers (i.e., new pressure ulcers) | Aged care and palliative care settings | Risk Ratio (RR): 1.33 [0.62, 2.88] | 36 (1) | Very low certainty |
|  |  | polyurethane foam dressing vs hydrogel dressing | Quality of life | Palliative care unit | Risk Ratio (RR): 0.33 [0.01, 7.65] | 34 (1) | Very low certainty |
|  | Westby 2017 (16) | Advanced dressing vs basic dressing | Time to complete healing | Long-term care settings | Hazard Ratio (HR): 1.13 [0.42, 3.00] | 36 (1) | Not assessed |
|  |  | Hydrocolloid +/- alginate vs ineligible: radiant heat | Time to complete healing | Long-term care settings | Hazard Ratio (HR): 0.64[0.23, 1.77] | 41 (1) | Not assessed |
|  |  | Hydrogel vs hydrocolloid | Time to complete healing | Long-term care settings | Hazard Ratio (HR): 1.75 [1.00, 3.05] | 43 (1) | Not assessed |
|  |  | polyurethane foam dressing vs hydrogel dressing | Time to complete healing | Palliative care unit | Compared to the hydrogel dressings, foam dressings were associated with an increased number of treatment days MD: 5.67 days, (95% CI -4.03 to 15.37) | 34 (1) | Very low certainty |
|  |  | Polyurethane, silver and ibuprofen-releasing foam dressing vs gauze, saline-soaked gauze, low-adherence dressing secured by a vapour-permeable film | Time to complete healing | Community dwelling | foam dressings were associated with a decreased time to complete healing MD -35.8 days, (95% CI -56.77 to -14.83) | 95 (1) | Very low certainty |
|  |  | Protease-modulating dressing vs advanced dressing | Time to complete healing | Long-term care settings | Hazard Ratio (HR): 1.34 [0.67, 2.65] | 65 (1) | Not assessed |
|  | Hao 2017 (3) | Topical phenytoin vs hydrocolloid dressing | Time to complete healing | Long-term care settings | phenytoin group was 35.3 ± 14.3 days (mean ± standard deviation (SD)) days compared with 51.8 ± 19.6 (mean ± SD) days for the hydrocolloid dressing (Duoderm) group | 28 (1) | Not assessed |
|  |  | Topical phenytoin vs placebo/alternative treatments/no therapy | Adverse events | Any setting | No studies found | NA | NA |
|  |  | Topical phenytoin vs triple antibiotic ointment | Time to complete healing | Long-term care settings | phenytoin group was 35.3 ±14.3 days (mean ± standard deviation (SD)) days compared with 53.8 ± 8.5 (mean ± SD) days for triple antibiotic ointment group | 26 (1) | Not assessed |

## Appendix 9. Detailed description of the results including the most effective interventions.

### Risk factors for pressure injuries

Ferris et al (2) included nine non-randomized studies (63,846 participants) that reported on risk factors for pressure ulcer development. The effect sizes were not reported for each risk factor. Instead, the authors reported the frequency of studies that reported each risk factor to be statistically significant (*p* < 0.05). Poor physical activity or activity of daily living ability (odds ratio (OR): 1.40), advanced age (> 62.9 yrs), Waterlow score (used to assess the risk for the development of pressure ulcer) > 15 predicted 95% of pressure ulcers, and longer duration of stay were identified in more than one article.

### Prevalence of pressure injuries

Ferris et al (2) pooled 11 non-randomized studies (63,846 participants) and reported an overall prevalence of 12.4% (range: 9.9% - 54.7%). The wide range of prevalence was attributed to differences in study settings and patient demographics. The authors of the review identified a single primary study (18) that reported the prevalence disaggregated by setting, revealing a prevalence of 6.9% - 16.2% in nursing homes, 13.8% - 19% among hospital inpatients, and 10.2% - 11% in home care settings. Another review (14) of 30 studies encompassing 355,784 individuals estimated a prevalence of pressure ulcers among older adults residing in nursing homes to be 11.6% (95% CI: 9.6 to 13.7%). Removal of stage I ulcers dropped the prevalence to 7.2% (95% CI: 6.2 to 8.3%) across 15 studies with a larger combined sample of 5,421,798 residents.

### Prevention of pressure injuries

#### Nutritional interventions

One high quality review (6) of Randomized Controlled Trials (RCT)s assessed the impact of three different nutritional combinations and diets for pressure ulcer prevention. Interventions included amino acids such as arginine, protein supplementation, and disease-specific diets. The RCTs (1 to 4 studies) were mainly conducted in hospital and long-term care settings and included 34 to 4,264 participants. The diets resulted in little to no effect in reducing the incidence of pressure ulcers. Among them, protein supplements had reduction in incidence (Risk ratio (RR): 0.75; 95% CI: 0.49, 1.14, n = 4264; low certainty) but was non-significant.

#### Repositioning strategies

One high quality review (17) of three relevant RCTs assessed the impact of repositioning frequencies and different tilt angles to prevent pressure ulcers in long term care residents. While more frequent repositioning was generally associated with lower incidence of pressure ulcers, the findings were inconsistent with no clear trend emerging. For example, one RCT demonstrated that repositioning the patient every 3hrs compared to every 4hrs had the greatest reduction in incidence (Risk ratio (RR): 0.20; 95% CI: 0.04, 0.92, n = 632; low certainty). In contrast, two RCTs showed that repositioning every 2hrs compared to every 3hrs produced highly variable results from 0.90 to 4.6 (very low certainty). The overall certainty of evidence was rated down due to serious limitations in design and imprecision (low number of events and wide confidence intervals). Another single RCT of 213 residents assessed the impact of a 30° tilt every 3hrs compared to 6-hourly 90° tilt (overnight) and a significant reduction in the risk of developing pressure ulcers (RR: 0.27, 95%CI: 0.08, 0.91; very low certainty).

The review noted that no studies were found that assessed adverse events from repositioning regimens or its impact on quality of life.

#### Support surfaces

Five reviews (5,8,11–13) (four high quality (8,11–13)) evaluated the effects of different support surfaces on preventing pressure ulcer development. There was moderate to high overlap between the three reviews conducted by Shi et al (11–13) and McInnes et al (8) as they addressed very similar questions with each review by Shi et al. focusing on a particular type of support surface. Herein, we describe the findings from the latest, most compressive and highest-quality reviews (8,11).

McInnes et al (8) evaluated more broadly the impact of support surfaces. They described 13 comparisons for different support surfaces including alternating pressure air surfaces, gel mattresses and continuous low-pressure (CLP) support surfaces. All the comparisons were made from single RCTs conducted in nursing homes and long-term care settings. Overall, the incidence of pressure ulcers (i.e., new pressure ulcers) ranged from RR: 0.42 (95%CI: 0.18, 0.96) to 2.25 (95%CI: 1.01, 5.02), depending on the type of support surface. The certainty of evidence was not assessed in these trials.

Shi et al (11) conducted a meta-analysis of three RCTs assessing alternating pressure (active) air surfaces compared to foam surfaces in a total of 2171 residents living in community inpatient facilities, Long-term care settings and nursing homes and demonstrated a RR of 0.79 (95%CI: 0.60, 1.03, I^2^ = 0%; low certainty). However, alternating pressure (active) air surfaces were not associated with a clear decrease in time to pressure ulcer development or better quality of life.

#### Organizational strategies

Two reviews high quality reviews included organization strategies including using normal and enhanced multidisciplinary teams (4) to prevent pressure ulcers and training and monitoring approaches (10) for nurses to implement when caring for long-term care residents for pressure ulcer prevention. Both reviews were informed by single RCTs while Joyce et al also included a quasi-experimental cluster study. The use of enhanced multidisciplinary teams was assessed in a trial involving 161 residents in long-term care settings. The intervention included advanced practice nurses (APNs) who provided outreach to long-term care facilities and were linked to a hospital-based expert wound care team. However, it remains unclear whether such teams reduce the incidence of pressure ulcers compared to usual care (HR 1.12, 95% CI: 0.74, 1.68; very low certainty) or improve time to complete healing (HR: 1.48, 95% CI: 0.79, 2.78; very low certainty). Similarly, the effects of training care providers in residential homes (RR: 0.63, 95%CI: 0.37, 1.05; very low certainty) and implementing monitoring strategies for residents (RR: 1.93, 95%CI: 0.96, 3.88; very low certainty) showed similar uncertainties regarding their impact on pressure ulcer prevention. The evidence was downgraded for serious limitations due to performance bias, detection bias, outcome reporting bias, and imprecision.

### Treatment of pressure injuries

#### Electrical stimulation

A single high quality review (1) of 12 relevant RCTs assessed the effects of electrical stimulation compared to placebo or sham stimulation to treat pressure ulcers. A trial of 38 participants in rehabilitation and geriatric care found no significant difference in time to complete healing (Hazard Ratio [HR]: 1.05, 95% CI: 0.44, 2.51; certainty not assessed). It is uncertain whether electrical stimulation reduces the surface area of pressure ulcers, with data from eight trials including 336 participants showing a mean difference ranging from -0.90 cm^2^ to 5.55 cm^2^ (very low certainty due serious risk of bias and imprecision). Adverse events were reported in four RCTs but they were attributed to the participants’ comorbidities rather than the intervention. Five RCTs reported no events and one RCT reported that 15% of the participants in the electrical stimulation group reported minor tingling post intervention (low certainty).

#### Nutritional interventions

One high quality review (6) of RCTs assessed the impact of nine different nutritional combinations and diets for pressure ulcer treatment. Interventions included amino acids such as arginine, protein supplementation, and specific formulations of micronutrients such as zinc, and antioxidants. The RCTs (1 to 3 studies) were mainly conducted in hospital and long-term care settings and included 16 to 283 participants.

Nutritional interventions did not result in improvement of the rate of pressure ulcer healing. Three different combinations of collagen, protein supplements and micronutrients resulted in a non-statistically significant decrease in pressure ulcer surface area (~2 cm^2^) (very low to moderate certainty). The use of an ornithine alpha-ketoglutarate supplements showed no effect on ulcer surface area. A meta-analysis of two RCTs on arginine and micronutrients compared to a standard hospital diet reported a reduction in ulcer surface area with a mean difference (MD) of −15.80 cm^2^ (95% Confidence Intervals (CI): -25.11, -6.48); I^2^ = 0%, n = 231; low certainty). Overall, none of the diets were associated with gastrointestinal adverse events. The overall certainty of evidence was rated as low to very low, mainly due high risk of bias and serious imprecision.

#### Organizational strategies

One high-quality review (4) included a single eligible RCT of 181 residents examining enhanced multidisciplinary teams (EMDTs) versus usual care in long-term care settings. EMDTs included outreach by advanced practice nurses and hospital-linked wound care specialists. There were uncertain effects on time to complete healing (HR: 1.48, 95% CI: 0.79, 2.78; very low certainty), hospitalization (HR: 1.20, 95% CI: 0.62, 2.36; very low certainty), and emergency department visits (HR: 1.30, 95% CI: 0.58, 2.90; very low certainty). Quality of life, measured using Q5D utilities, also showed minimal and uncertain benefit (MD: 0.03, 95% CI: –0.03 to 0.09; very low certainty). Additionally, a comparison of multidisciplinary wound care teams to usual care revealed improved time to healing (HR: 1.73, 95% CI: 1.20, 2.50; very low certainty), though confidence remains limited due to serious methodological limitations including imprecision and risk of bias.

#### Topical and dressing interventions

Four high-quality reviews assessed the effects of topical agents and dressings to prevent (9) and treat (3,15,16) pressure ulcers. There was moderate overlap (8.3%) between the reviews, particularly between the two older reviews (15,16). The most recent review (9) which focused on prevention, concluded that it is uncertain whether the use of topical creams reduces the incidence of pressure ulcer development compared to placebo (low certainty). One notable finding was the use of dimethyl sulfoxide (DMSO) cream among 79 nursing home residents, which was associated with an increased risk of pressure ulcer development (RR: 1.99; 95%CI: 1.1, 3.57; very low certainty) compared to placebo. Adverse events were associated with the topical use of fatty acids compared to olive oil in 247 nursing home residents (RR: 2.22, 95%CI: 0.2, 24.37; low certainty). No studies assessed the impact of these agents on quality of life.

Eight dressings were evaluated for their impact on treating pressure ulcers (15,16). Polyurethane foam dressings were compared to hydrogel dressings in 34 individuals in palliative care units. No evidence was found for reduced pressure ulcer incidence, improved time to complete healing or quality of life (very low certainty). Hydrocellular, hydropolymer, and polyurethane foam dressings were compared with hydrocolloid dressings in 198 patients in aged care and palliative care settings. The results showed no significant difference in pressure ulcer outcomes (RR: 0.85, 95% CI: 0.54 to 1.34; I² = 5.73%; very low certainty). The certainty of evidence was reduced due to serious limitations in study design and implementation as well as serious imprecision. These aforementioned types of dressing were not associated with any adverse events.

## Appendix 10. Risk of bias of primary studies in the included systematic reviews.

| Review ID | Primary study ID | **Random sequence generation (n*)%** | **Concealed allocation (n)%** | **Blinding of participants (n)%** | **Blinding of personnel (n)%** | **Blinding of outcome assessment (n)%** | **Incomplete outcome data (n)%** | **Selective reporting (n)%** |
| --- | --- | --- | --- | --- | --- | --- | --- | --- |
| Arora 2020 | Adunksy 2005 | low | low | low | low | low | high | low |
|  | Asbjornsen 1990 | unclear | unclear | low | unclear | low | high | low |
|  | Carley 1985 | unclear | unclear | high | high | unclear | low | low |
|  | Feeder 1991 | unclear | unclear | low | low | low | high | high |
|  | Franek 2011 | low | low | high | high | low | low | low |
|  | García-Pérez 2018 | low | high | high | high | low | low | low |
|  | Gentzkow 1991 | unclear | unclear | low | low | unclear | high | low |
|  | Kloth 1988 | low | low | low | low | low | low | low |
|  | Polak 2016a | unclear | low | low | high | low | high | low |
|  | Polak 2016b | low | low | high | high | unclear | low | low |
|  | Polak 2017 | low | low | low | high | low | low | low |
|  | Wood 1993 | unclear | unclear | low | low | low | low | low |
| Gillespie 2020 | Bergstrom 2013 | low | low | high | high | low | unclear | low |
|  | Defloor 2005 | low | unclear | high | high | high | high | low |
|  | Moore 2011 | low | low | high | high | high | low | low |
| Hao 2017 | Rhodes 2001 | unclear | unclear | unclear | unclear | unclear | high | low |
| Joyce 2018 | Stern 2014 | low | low | high | high | low | high | low |
|  | Vu 2007 | high | high | high | high | unclear | high | unclear |
| McInnes 2015 | Conine 1993 | unclear | unclear | unclear | unclear | unclear | low | low |
|  | Conine 1994 | unclear | unclear | low | low | low | low | low |
|  | Geyer 2001 | low | low | low | low | low | low | low |
|  | Hampton 1997 | unclear | unclear | unclear | unclear | unclear | unclear | unclear |
|  | Lazzara 1991 | low | unclear | unclear | unclear | unclear | unclear | low |
|  | Nixon 2006 | low | low | high | high | high | low | low |
|  | Ricci 2013 | low | low | unclear | unclear | unclear | unclear | unclear |
|  | Tymec 1997 | low | unclear | unclear | unclear | unclear | unclear | low |
|  | Van Leen 2011 | unclear | unclear | low | low | low | unclear | low |
|  | Vyhlidal 1997 | low | unclear | unclear | unclear | unclear | low | low |
| Moore 2018 | Diaz-Valenzuela 2014 | low | unclear | low | low | low | low | low |
|  | Green 1974 | unclear | unclear | low | low | low | high | low |
|  | Houwing 2008 | low | unclear | low | low | low | low | low |
|  | Nakagami 2007 | unclear | unclear | high | high | high | low | low |
|  | Smith 1985 | unclear | unclear | low | low | low | low | low |
|  | Lupianez-Perez 2015 | low | low | low | low | low | low | unclear |
|  | Otero 2017 | low | unclear | high | high | low | high | low |
| Porter-Armstrong 2018 | James 1998 | unclear | unclear | high | high | unclear | high | unclear |
| Shi 2021 | Nixon 2019 | low | low | high | high | low | low | low |
|  | Ballard 1997 | unclear | unclear | unclear | unclear | unclear | high | low |
|  | Beeckman 2019 | low | unclear | high | high | high | low | low |
|  | Grindley 1996 | low | low | unclear | unclear | unclear | unclear | low |
|  | Hampton 1997 | unclear | unclear | unclear | unclear | unclear | unclear | unclear |
|  | Nixon 2006 | low | low | high | high | unclear | low | low |
|  | Phillips 1999 | unclear | unclear | unclear | unclear | unclear | unclear | high |
|  | Rosenthal 2003 | low | unclear | unclear | unclear | unclear | unclear | low |
|  | Sauvage 2017 | low | unclear | high | high | high | low | low |
| Shi 2021b foam surfaces | Rosenthal 2003 | low | unclear | unclear | unclear | unclear | unclear | low |
|  | van Leen 2013 | low | unclear | unclear | unclear | unclear | low | low |
|  | van Leen 2011 | unclear | unclear | unclear | unclear | low | low | low |
|  | Sauvage 2017 | low | unclear | high | high | high | low | low |
| Shi 2021a reactive air | Beeckman 2019 | low | unclear | high | high | high | low | low |
|  | Lazzara 1991 | low | unclear | unclear | unclear | unclear | unclear | low |
|  | van Leen 2013 | low | unclear | unclear | unclear | unclear | low | low |
|  | van Leen 2011 | unclear | unclear | unclear | unclear | low | low | low |
| Westby 2017 | Brod 1990 | unclear | unclear | high | high | high | low | low |
|  | Graumlich 2003 | low | low | low | low | low | low | low |
|  | Payne 2009 | unclear | unclear | unclear | unclear | unclear | high | low |
|  | Thomas 2005 | unclear | unclear | high | high | high | high | low |
| Walker 2017 | Bale 1997 | unclear | unclear | unclear | unclear | unclear | high | low |
|  | Bale 1998 | unclear | unclear | unclear | unclear | high | low | unclear |
|  | Banks 1994a | low | low | unclear | unclear | unclear | unclear | unclear |
|  | Meaume 2003 | low | low | high | high | high | low | low |
|  | Payne 2009 | unclear | high | high | high | high | low | unclear |
|  | Seeley 1999 | low | low | high | high | high | low | low |
|  | Sopata 2002 | unclear | unclear | unclear | unclear | high | low | low |
|  | Souliotis 2016 | unclear | low | high | high | high | unclear | unclear |
|  | Thomas 1997 | unclear | high | high | high | high | low | unclear |

| Review ID | primary study ID | **Bias from randomization process** | **Bias from deviation from intended intervention** | **Bias due to missing outcome data** | **Bias in measurement of the outcome** | **Bias in selection of the reported result** | **Overall** |
| --- | --- | --- | --- | --- | --- | --- | --- |
| Langer 2024 | Anbar 2014 | low | unclear | low | unclear | low | unclear |
|  | Banks 2016 | unclear | unclear | high | unclear | low | high |
|  | Benati 2001 | unclear | unclear | unclear | unclear | unclear | unclear |
|  | Botella Carretero 2008 | low | unclear | low | unclear | unclear | unclear |
|  | Bourdel Marchasson 2000 | high | unclear | low | high | unclear | high |
|  | Brewer 1967 | low | low | unclear | low | unclear | unclear |
|  | Cereda 2009 | high | low | low | low | unclear | high |
|  | Cereda 2015 | low | low | low | low | low | low |
|  | Chernoff 1990 | unclear | unclear | low | unclear | unclear | unclear |
|  | Craig 1998 | unclear | unclear | unclear | unclear | unclear | unclear |
|  | Desneves 2005 | high | low | low | low | unclear | high |
|  | Ek 1991 | unclear | high | high | unclear | unclear | high |
|  | Hartgrink 1998 | unclear | low | unclear | unclear | unclear | unclear |
|  | Leigh 2012 | low | high | unclear | low | unclear | high |
|  | Meaume 2009 | low | low | low | unclear | unclear | unclear |
|  | Miu 2021 | high | unclear | unclear | unclear | low | high |
|  | Norris 1971 | unclear | unclear | high | low | unclear | high |
|  | Ohura 2011 | high | high | high | unclear | unclear | high |
|  | Pouyssegur 2015 | high | high | high | high | unclear | high |
|  | Sugihara 2018 | unclear | low | low | low | low | unclear |
|  | Taylor 1974 | high | unclear | low | low | unclear | high |
|  | Ter Riet 1995 | unclear | low | low | low | unclear | unclear |
|  | Theilla 2012 | unclear | unclear | low | unclear | low | unclear |
|  | Van Anholt 2010 | unclear | low | unclear | low | low | unclear |
|  | Wong 2014 | low | unclear | low | low | low | unclear |
|  | Yamanaka 2017 | low | low | low | low | unclear | unclear |

|  | Author  (year) | External validity | | | | Internal validity | | | | | |
| --- | --- | --- | --- | --- | --- | --- | --- | --- | --- | --- | --- |
| Review ID |  | 1. Was the study’s target population a close representation of the national population in relation to relevant variables? | 2. Was the sampling frame a  true or close representation of the target population? | 3. Was some form of random selection used to select the sample, OR, was a census undertaken? | 4. Was the likelihood of non-response bias minimal? | 5. Were data collected directly from the subjects (as opposed to a proxy)? | 6. Was an acceptable case definition used in the study? | 7. Was the study instrument that measured the parameter of interest shown to have reliability and validity (if necessary)? | 8. Was the same mode of data collection used for all subjects? | 9. Was the length of the shortest prevalence period for the parameter of interest appropriate? | 10. Were the numerator(s) and denominator(s) for the parameter of interest appropriate? |
| Sugathapala 2023 | Bååth 2014 | Y | Y | Y | N | Y | Y | N | Y | Y | Y |
|  | Banks 2010 | N | N | N | N | Y | Y | N | Y | Y | Y |
|  | Barrois 2018 | Y | Y | N | N | Y | Y | N | N | Y | N |
|  | Bates- Jensen 2003 | N | N | N | Y | N | N | N | Y | Y | Y |
|  | Bates- Jensen 2009 | N | N | N | Y | Y | Y | Y | Y | Y | Y |
|  | Bates- Jensen 2017  Bates- Jensen 2018 | N | N | N | Y | Y | Y | Y | Y | Y | Y |
|  | Bliss 2015 | Y | Y | Y | Y | N | N | N | Y | Y | Y |
|  | Carryer 2017 | Y | Y | Y | Y | Y | Y | N | Y | Y | Y |
|  | Casimiro 2002 | Y | Y | N | N | Y | N | N | Y | Y | Y |
|  | Chacon 2009 | N | Y | Y | Y | Y | Y | N | Y | Y | Y |
|  | Courvosier 2018 | N | Y | Y | Y | Y | Y | N | Y | Y | Y |
|  | Demarre 2015 | N | N | N | N | N | Y | N | Y | Y | Y |
|  | de Souza 2010 | N | N | N | N | Y | Y | N | Y | Y | Y |
|  | Dijkstra 2015 | Y | N | N | N | N | N | N | Y | Y | Y |
|  | Doupe 2016 | N | Y | Y | Y | N | N | N | Y | Y | Y |
|  | Edwards 2017 | Y | N | Y | Y | Y | Y | Y | Y | Y | Y |
|  | Gerardo 2009 | N | Y | Y | N | N | Y | N | Y | Y | Y |
|  | Gunningberg 2004 | N | Y | Y | N | Y | Y | Y | Y | Y | Y |
|  | Hahnel 2017 | Y | Y | N | N | Y | N | Y | Y | Y | Y |
|  | Harms 2014 | Y | Y | Y | Y | N | N | N | Y | Y | Y |
|  | Hartmann 2016 | N | N | N | N | N | N | N | Y | Y | Y |
|  | Hernandez-Martínez-Esparza 2021 | N | N | N | Y | Y | Y | N | Y | Y | Y |
|  | Jorgensen 2018 | N | Y | Y | Y | N | N | N | Y | Y | Y |
|  | Kim 2017 | Y | Y | Y | Y | N | N | N | Y | Y | Y |
|  | Kottner 2010 | Y | Y | N | Y | Y | Y | N | Y | Y | Y |
|  | Kwong 2009 | N | N | N | N | Y | Y | Y | Y | Y | N |
|  | Lahmann 2010a | Y | N | N | Y | Y | Y | N | Y | Y | Y |
|  | Lahmann 2015 | Y | N | N | N | Y | Y | Y | Y | Y | Y |
|  | Li 2011 | Y | N | Y | N | N | N | N | Y | Y | Y |
|  | Lichterfeld-Kottner 2020 | Y | N | N | N | Y | N | N | Y | Y | Y |
|  | Meesterberends 2013 | Y | Y | N | N | Y | N | N | Y | Y | Y |
|  | Moore 2012 | N | Y | N | N | Y | Y | N | Y | Y | Y |
|  | Nakashima 2018 | N | Y | N | N | Y | Y | Y | Y | Y | Y |
|  | Okuwa 2006 | N | Y | Y | Y | Y | Y | N | Y | Y | Y |
|  | Palese 2020 | N | Y | N | N | N | N | N | Y | Y | Y |
|  | Rasero 2015 | N | Y | Y | Y | Y | N | N | Y | Y | Y |
|  | Righi 2020 | N | N | N | N | Y | N | N | Y | Y | Y |
|  | Stolt 2019 | N | N | N | N | Y | N | N | Y | Y | Y |
|  | Suzuki 2021 | Y | Y | Y | N | Y | N | N | Y | Y | Y |
|  | Tannen 2006 | N | Y | Y | N | Y | Y | Y | Y | Y | Y |
|  | Tannen 2008 | N | N | Y | N | Y | Y | N | Y | Y | Y |
|  | Tannen 2009 | Y | Y | N | Y | Y | Y | Y | Y | Y | Y |
|  | Thein 2010 | N | Y | Y | Y | N | N | N | N | Y | Y |
|  | Trinks 2018 | Y | Y | Y | Y | N | N | N | Y | Y | Y |
|  | Van Gaal 2014 | N | N | N | N | Y | Y | N | Y | Y | Y |
|  | VanGilder 2017 | Y | N | N | N | Y | Y | Y | Y | Y | Y |
|  | Wei 2021 | N | Y | Y | Y | Y | Y | Y | Y | Y | Y |

| Study | Random Assignment | Participant Blinding | Allocation Concealed | Withdrawals Described | Assessor Blinded | Groups Comparable | Identical Treatment | Same Outcome Measure | Reliable Outcome Measure | Appropriate Stats | Total quality score (out of 10) |
| --- | --- | --- | --- | --- | --- | --- | --- | --- | --- | --- | --- |
| Appraisal of Randomised controlled trials (n = 10) | | | | | | | | | | | |
| Bergstrom et al. 2014 | Y | N | Y | N | Y | Y | Y | Y | a | Y | 8 |
| Brienza et al. 2010 | Y | N | N | N | Y | Y | Y | Y | a | Y | 7 |
| van Leen et al. 2011 | Y | N | N | Y | N | N | Y | Y | Y | N | 5 |
| van Leen et al. 2013 | Y | N | N | N | n/a | Y | Y | Y | Y | Y | 6 |
| Moore et al. 2011 | Y | N | Y | N | Y | Y | Y | Y | Y | Y | 8 |
| Pouyssegur et al. 2015 | Y | N | N | N | N | Y | Y | Y | Y | Y | 6 |
| Ricci et al. 2013 | Y | N | Y | n/a | ? | Y | Y | Y | N | Y | 6 |
| Shannon et al. 2012 | Y | N | N | N | N | Y | Y | Y | a | Y | 6 |
| Stern et al. 2014 | Y | N | Y | N | Y | Y | Y | Y | Y | Y | 8 |
| Vanderwee et al. 2006 | Y | N | N | n/a | N | Y | Y | Y | Y | Y | 6 |
| Appraisal of cohort with control/case–control studies (n = 3) | | | | | | | | | | | |
|  | Representative Sample | Similar Disease Stage | Bias Minimised in Selection | Confounders Addressed | Objective Outcome Assessment | Sufficient Follow-up | Withdrawals Described | Reliable Outcome Measure | Appropriate Stats | Total quality score (out of 10) | |
| Fossum et al. 2011 | Y | Y | N | N | Y | Y | N | Y | Y | 6 | |
| van Leen  et al.  2014 | Y | Y | Y | Y | Y | Y | n/a | Y | Y | 8 | |
| Olsho et  al. 2014 | Y | N | N | Y | Y | Y | Y | Y | Y | 7 | |

**References**

1. Arora M, Harvey LA, Glinsky JV, Nier L, Lavrencic L, Kifley A, et al. Electrical stimulation for treating pressure ulcers. Cochrane Database Syst Rev. 2020;1(1):Cd012196.

2. Ferris A, Price A, Harding K. Pressure ulcers in patients receiving palliative care: A systematic review. Palliat Med [Internet]. 2019;33(7):770–82. Available from: https://login.proxy.bib.uottawa.ca/login?url=http://ovidsp.ovid.com/ovidweb.cgi?T=JS&CSC=Y&NEWS=N&PAGE=fulltext&D=med16&DO=10.1177%2f0269216319846023

3. Hao XY, Li HL, Su H, Cai H, Guo TK, Liu R, et al. Topical phenytoin for treating pressure ulcers. Cochrane Database Syst Rev. 2017;2(2):Cd008251.

4. Joyce P, Moore ZE, Christie J. Organisation of health services for preventing and treating pressure ulcers. Cochrane Database Syst Rev. 2018;12(12):Cd012132.

5. Junkin J, Gray M. Are pressure redistribution surfaces or heel protection devices effective for preventing heel pressure ulcers? J Wound Ostomy Cont Nurs. 2009;36(6):602–8.

6. Langer G, Wan CS, Fink A, Schwingshackl L, Schoberer D. Nutritional interventions for preventing and treating pressure ulcers. Cochrane Database Syst Rev [Internet]. 2024;2024(2). Available from: https://www.scopus.com/inward/record.uri?eid=2-s2.0-85185137637&doi=10.1002%2f14651858.CD003216.pub3&partnerID=40&md5=206361be8d7b8388867a57cbcee1e560

7. Mäki-Turja-Rostedt S, Stolt M, Leino-Kilpi H, Haavisto E. Preventive interventions for pressure ulcers in long-term older people care facilities: A systematic review. J Clin Nurs. 2019 Jul;28(13–14):2420–42.

8. McInnes E, Jammali-Blasi A, Bell-Syer SEM, Dumville JC, Middleton V, Cullum N. Support surfaces for pressure ulcer prevention. Cochrane Database Syst Rev. 2015 Sep 3;2015(9):CD001735.

9. Moore ZE, Webster J. Dressings and topical agents for preventing pressure ulcers. Cochrane Database Syst Rev. 2018;12(12):Cd009362.

10. Porter-Armstrong AP, Moore ZE, Bradbury I, McDonough S. Education of healthcare professionals for preventing pressure ulcers. Cochrane Database Syst Rev. 2018;5(5):Cd011620.

11. Shi C, Dumville JC, Cullum N, Rhodes S, Jammali-Blasi A, McInnes E. Alternating pressure (active) air surfaces for preventing pressure ulcers. Cochrane Database Syst Rev. 2021;5(5):Cd013620.

12. Shi C, Dumville JC, Cullum N, Rhodes S, Leung V, McInnes E. Reactive air surfaces for preventing pressure ulcers. Cochrane Database Syst Rev. 2021;5(5):Cd013622.

13. Shi C, Dumville JC, Cullum N, Rhodes S, McInnes E. Foam surfaces for preventing pressure ulcers. Cochrane Database Syst Rev. 2021;5(5):Cd013621.

14. Sugathapala RDUP, Latimer S, Balasuriya A, Chaboyer W, Thalib L, Gillespie BM. Prevalence and incidence of pressure injuries among older people living in nursing homes: A systematic review and meta-analysis. Int J Nurs Stud. 2023 Dec;148:104605.

15. Walker RM, Gillespie BM, Thalib L, Higgins NS, Whitty JA. Foam dressings for treating pressure ulcers. Cochrane Database Syst Rev [Internet]. 2017;(10). Available from: https://login.proxy.bib.uottawa.ca/login?url=http://ovidsp.ovid.com/ovidweb.cgi?T=JS&CSC=Y&NEWS=N&PAGE=fulltext&D=coch&AN=00075320-100000000-09740

16. Westby MJ, Dumville JC, Soares MO, Stubbs N, Norman G. Dressings and topical agents for treating pressure ulcers. Cochrane Database Syst Rev. 2017;6(6):Cd011947.

17. Gillespie BM, Walker RM, Latimer SL, Thalib L, Whitty JA, McInnes E, et al. Repositioning for pressure injury prevention in adults. Cochrane Database Syst Rev. 2020;6(6):Cd009958.

18. Carlsson ME, Gunningberg L. Predictors for Development of Pressure Ulcer in End-of-Life Care: A National Quality Register Study. J Palliat Med. 2017 Jan;20(1):53–8.
